# Genome Wide Analysis Reveals Zic3 Interaction with Distal Regulatory Elements of Stage Specific Developmental Genes in Zebrafish

**DOI:** 10.1371/journal.pgen.1003852

**Published:** 2013-10-31

**Authors:** Cecilia L. Winata, Igor Kondrychyn, Vibhor Kumar, Kandhadayar G. Srinivasan, Yuriy Orlov, Ashwini Ravishankar, Shyam Prabhakar, Lawrence W. Stanton, Vladimir Korzh, Sinnakaruppan Mathavan

**Affiliations:** 1 Human Genetics, Genome Institute of Singapore, Singapore, Singapore; 2 Fish Developmental Biology, Institute of Molecular and Cell Biology, Singapore, Singapore; 3 Computational and Mathematical Biology, Genome Institute of Singapore, Singapore, Singapore; 4 Functional Genomics Core, Singapore Immunology Network, Singapore, Singapore; 5 Institute of Cytology and Genetics SB RAS, Novosibirsk, Russia; 6 Stem Cell and Developmental Biology, Genome Institute of Singapore, Singapore, Singapore; Johns Hopkins University, United States of America

## Abstract

Zic3 regulates early embryonic patterning in vertebrates. Loss of Zic3 function is known to disrupt gastrulation, left-right patterning, and neurogenesis. However, molecular events downstream of this transcription factor are poorly characterized. Here we use the zebrafish as a model to study the developmental role of Zic3 *in vivo*, by applying a combination of two powerful genomics approaches – ChIP-seq and microarray. Besides confirming direct regulation of previously implicated Zic3 targets of the Nodal and canonical Wnt pathways, analysis of gastrula stage embryos uncovered a number of novel candidate target genes, among which were members of the non-canonical Wnt pathway and the neural pre-pattern genes. A similar analysis in *zic3*-expressing cells obtained by FACS at segmentation stage revealed a dramatic shift in Zic3 binding site locations and identified an entirely distinct set of target genes associated with later developmental functions such as neural development. We demonstrate cis-regulation of several of these target genes by Zic3 using *in vivo* enhancer assay. Analysis of Zic3 binding sites revealed a distribution biased towards distal intergenic regions, indicative of a long distance regulatory mechanism; some of these binding sites are highly conserved during evolution and act as functional enhancers. This demonstrated that Zic3 regulation of developmental genes is achieved predominantly through long distance regulatory mechanism and revealed that developmental transitions could be accompanied by dramatic changes in regulatory landscape.

## Author Summary

The Zic3 transcription factor regulates early embryonic patterning, and the loss of its function leads to defects in left-right body asymmetry. Previous studies have only identified a small number of Zic3 targets, which renders the molecular mechanism underlying its activity insufficiently understood. Utilizing two genomics technologies, next generation sequencing and microarray, we profile the genome-wide binding sites of Zic3 and identified its target genes in the developing zebrafish embryo. Our results show that Zic3 regulates its target genes predominantly through regulatory elements located far from promoters. Among the targets of Zic3 are the Nodal and Wnt pathways known to regulate gastrulation and left-right body asymmetry, as well as neural pre-pattern genes regulating proliferation of neural progenitors. Using enhancer activity assay, we further show that genomic regions bound by Zic3 function as enhancers. Our study provides a genome-wide view of the regulatory landscape of Zic3 and its changes during vertebrate development.

## Introduction

Early embryonic patterning is achieved through a process involving the determination of body axes and defining which cell types develop at each coordinate. The Zic family of transcription factors (TFs) is involved in such process [1]–[4]. Zic genes are the vertebrate homologues of the *odd-paired* gene, which is involved in the generation of segmental body plan in the *Drosophila* embryo [5], [6]. Although functions of Zic proteins partially overlap, their loss-of-function cause distinct phenotypes, suggesting unique roles in development [7], [8].

Of particular interest is ZIC3, which is linked to the heritable defects of the left-right internal organs placement (*situs inversus*) in humans [9]. Studies in animal models reveal the involvement of Zic3 the establishment of left-right (L-R) asymmetry [1], [10]–[12]. In *Xenopus*, Zic3 established left-sided expression of *Xnr1* and *Pitx2* [12], two determinants of internal organs asymmetry [13]–[15]. However, *zic3* is expressed symmetrically along the L-R axis in the *Xenopus* embryo and its loss-of-function (LOF) affects structures in which its expression was not detected [1], [12]. Results from several studies provided clues to the mechanism of L-R patterning by Zic3. First, Zic3 acts in organizer formation by inhibiting the canonical Wnt signaling pathway [16]. Second, Zic3 regulates gastrulation in mouse [1], [17]. Furthermore, studies in zebrafish revealed a correlation between convergence-extension (C-E) and L-R patterning defects in Zic3 LOF [10]. These suggest that Zic3 may regulate L-R patterning through its role in an earlier developmental event such as C-E.

Zic3 is one of the earliest TFs expressed in the neuroectoderm [3], [18]. Its expression is regulated by determinants of the early neural fate specification and dorsal-ventral (D-V) axis formation, including BMP, FGF, and Nodal signaling [3], [17], [19], [20]. The role of Zic3 in establishing neural cell fate was demonstrated through experiments in *Xenopus*, where its overexpression resulted in the expansion of the neuroectoderm and induction of neural and neural crest markers [18]. This led to the assumption that Zic3 activates the expression of proneural genes such as *Achaete-scute* homologs, *Neurogenin*, and *NeuroD* [2]. However, Zic3 lacks the ability to induce ectopic neuronal differentiation in the epidermis [18], which suggested the complex interaction between Zic3 and the proneural genes.

Increasing evidence has established the presence of long-distance interactions between TFs and their target genes [21]–[24]. This feature is especially true for TFs regulating specific functions outside of the core transcription machinery [25]–[27]. Therefore, an unbiased evaluation of binding sites throughout the whole genome would be a more comprehensive and biologically relevant method in the context of a developing organism. However, genomic approaches to study TFs *in vivo* are often limited by the quantity of available tissue sample. Furthermore, in mammalian systems, this problem is exacerbated by the short supply of embryos at early developmental stages. The zebrafish, with its unlimited supply of embryos and external development, substitutes for the inconveniences of a mammalian system. Its genome annotation is also the most complete among non-mammalian vertebrates and the expression of many genes are well-defined. This makes the zebrafish a robust model system for functional studies of vertebrate development.

To understand the developmental role of Zic3, we applied a genomic approach to identify genes directly regulated by Zic3. To capture genome-wide binding sites of Zic3, chromatin fragments bound by Zic3 were immunoprecipitated from gastrulating embryos at 8 hpf and *zic3* expressing cells were sorted from transgenics [21], [28] at 24 hpf and sequenced in-depth using ChIP-seq methodology. This provided unbiased coverage of Zic3 binding events during the period of gastrulation and segmentation. We used microarray expression profiling to characterize changes at the transcription level as a result of Zic3 LOF during gastrulation. In addition, we compared gene expression profiles of *zic3*-positive and -negative cells at 24 hpf to identify genes co-expressed with *zic3*. Combining binding site analysis and expression data, we demonstrated that Nodal and Wnt pathways are the main downstream targets of Zic3 during gastrulation, and show distinct pathways regulated by Zic3 in the dorsal neural tube at the end of segmentation. Finally, *in vivo* enhancer assay validated selected binding sites as developmental enhancers. Our results provide novel insights into the molecular mechanism underlying Zic3 regulation of developmental events during gastrulation and neural development, which ultimately results in the L-R patterning and neural fate specification and patterning.

## Results

### Genome-wide identification of Zic3 binding sites using ChIP-seq

**Figure 1:**
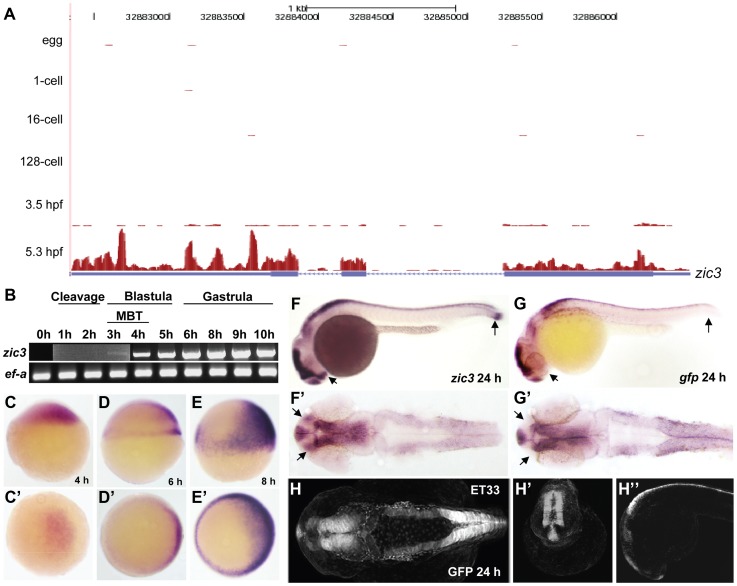
Early expression of *zic3* as detected by RNA-seq and RT-PCR. A, UCSC browser image showing RNA-seq reads (pink vertical histograms) at the *zic3* locus (in blue; tall boxes - exons; half-height boxes - UTRs; lines - introns; arrowheads - direction of transcription). B, RT-PCR detection of *zic3* transcripts in zebrafish embryo. Portion of image showing expression at 1 hpf to 3 hpf was enhanced to show weak band at 3 hpf. C, D and E, *zic3* expression in 4 hpf, 6 hpf, and 8 hpf embryos. Lateral view, dorsal to the right. C′, D′ and E′, animal pole view. F, G, *zic3* expression in wild-type and *gfp* in SqET33 embryos at 24 hpf. F′, G′, dorsal view. Note the absence of *gfp* expression from the olfactory bulb and presomitic mesoderm domains of the wild-type *zic3* expression (arrows). H–H″, GFP expression in live SqET33 embryos at 24 hpf. H, dorsal view, H′, frontal view, H″, lateral view.

The earliest *zic3* transcript was detected at 3 hpf (Fig. 1A,B), coinciding with the initiation of zygotic transcription during mid-blastula transition [29]. At 4 hpf *zic3* expression is restricted to dorsal blastoderm (Fig. 1C,C′), and is subsequently found in the dorsal neuroectoderm and marginal blastomeres (Fig. 1D, D′). To capture genome-wide Zic3 binding profile during zebrafish gastrulation, we performed ChIP-seq analysis at 8 hpf, a time coinciding with the beginning of neurogenesis [30]. At this time *zic3* is expressed largely in the dorsal neuroectoderm (prospective neural plate) and blastoderm margin (presumptive mesendoderm; Fig. 1E,E′; [3]). Hence, the interaction of Zic3 with its targets could be considered within a context of neural induction and mesendodermal development. Although neuroectoderm does not show any obvious morphological organization at this time, its anteroposterior patterning at the molecular level was shown by fate mapping studies [31] and *in vitro* explant assays [32], [33]. At 24 hpf *zic3* is expressed in the brain and dorsal spinal cord (Fig. 1F,F′). To identify Zic3 binding sites specifically in *zic3*-expressing cells, we performed ChIP-seq using sorted cells from transgenic line SqET33 [28], [34] at this stage. Since *gfp* expression in this line faithfully recapitulates *zic3* expression (Fig. 1G–H″), we considered GFP-positive cells as *zic3*-expressing cells and GFP-negative cells as non- *zic3*-expressing cells. However, it is worth to note that in SqET33 line at least one *zic3*-positive domain (presomitic mesoderm) does not express GFP. This suggests that a small fraction of non-neuronal *zic3*-expressing cells may be present in the GFP-negative pool of cells.

**Figure 2:**
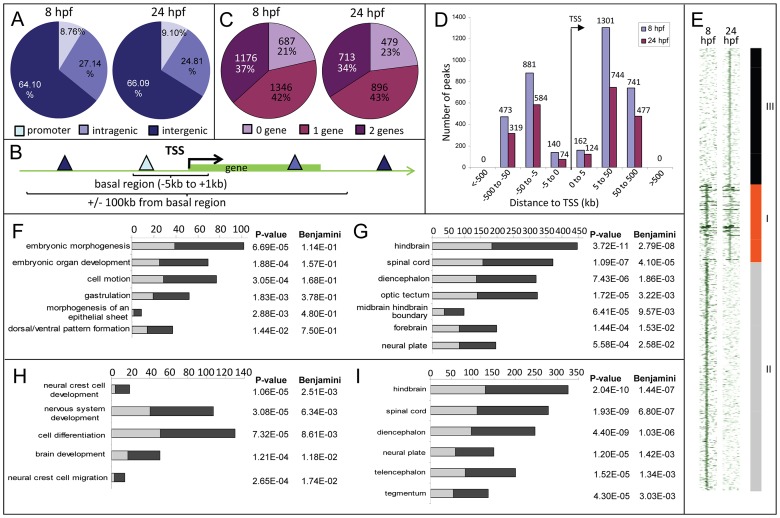
Distribution of Zic3 peaks as identified in ChIP-seq experiments according to GREAT algorithm. A, distribution of peaks located in promoter (within 5 kb upstream of TSS), intragenic, and intergenic regions. B, gene association rule of ‘basal plus 100 kb’ according to GREAT algorithm. C, percentage of peaks associated with none, one, or two genes based on the gene association rule in B. D, number of peaks present in each distance categories along the x-axis, with regards to TSS of associated gene. E, region map showing overlap between genomic locations of peaks in 8 hpf and 24 hpf datasets. F–I, list of biological processes and tissue specific expression terms enriched among Zic3-associated genes at 8 hpf (F, G) and 24 hpf (H, I) according to DAVID GO terms. Light and dark grey bars represent the expected and observed enrichments of functional categories indicated along the y-axis.

Sequencing of the 8 hpf ChIP sample generated 23,945,552 reads (11,037,221 or 46% were mapped to the zebrafish genome); the 24 hpf ChIP sample generated 23,083,504 reads (11,797,011 or 51% were mapped). We identified 3209 and 2088 Zic3 binding sites (hereafter referred to as peaks) with high significance value at 8 hpf (Table S13) and 24 hpf (Table S14), respectively. Interestingly, both datasets showed that only a small fraction (8.6% at 8 hpf and 4% at 24 hpf) of the peaks mapped to promoter regions (within 5 kb of transcription start site, TSS), while the rest were aligned to intragenic (26.8% at 8 hpf and 29% at 24 hpf) and intergenic (64.6% at 8 hpf and 67% at 24 hpf) regions (Fig. 2A). This suggested that Zic3 mainly acts via distal regulatory elements. To validate the ChIP-seq performance, we carried out quantitative PCR (qPCR) on randomly selected peaks from the 8 hpf dataset, five within promoter region and sixteen at regions outside of gene promoters. Taking a fold-change of 2 as a cutoff for positive enrichment, the qPCR analysis validated all but one peak tested (Table S1).

To determine the biological relevance of our data, we used the gene association rule ‘basal plus 100 kb extension’ according to GREAT algorithm [35] (Fig. 2B). Using this criterion, the number of peaks associated with either none, one, or two genes were evenly distributed in both 8 hpf and 24 hpf datasets (Fig. 2C). Distribution of the peaks relative to the TSS of genes associated with them showed strong bias towards regions beyond 5 kb of the TSS (Fig. 2D). In agreement with known Zic3 functions at 8 hpf [10], [16], [18], [36] functional categories enriched were embryonic morphogenesis, gastrulation, and dorsal/ventral pattern formation (2835 genes, Fig. 2F; Table S2). Enrichment was also observed for neural tissue-specific genes, predominantly expressed in the neuroectoderm at 8 hpf (Fig. 2G). In contrast, at 24 hpf, different categories were enriched (neural crest development and migration, nervous system development; Fig. 2H,I) in agreement with these events of neurodevelopment taking place at this stage [18], [37].

To identify the common regions bound by Zic3 as well as those unique to either developmental stage, we overlapped the 8 hpf and 24 hpf peaks (Fig. 2E). Taking the combined list of peaks from 8 hpf and 24 hpf, we performed clustering using ChIP-seq signals around the peaks. We found 937 regions bound by Zic3 at both stages (class I), 2729 regions bound only at 8 hpf (class II), and 1630 regions only at 24 hpf (class III). A clear distinction of functional categories was observed among genes associated with each individual class (Fig. S2), which reflect the shift of Zic3 function from regulating gastrulation at 8 hpf, to directing neurodevelopment at 24 hpf.

### Identification of Zic3 consensus binding motif

**Figure 3:**
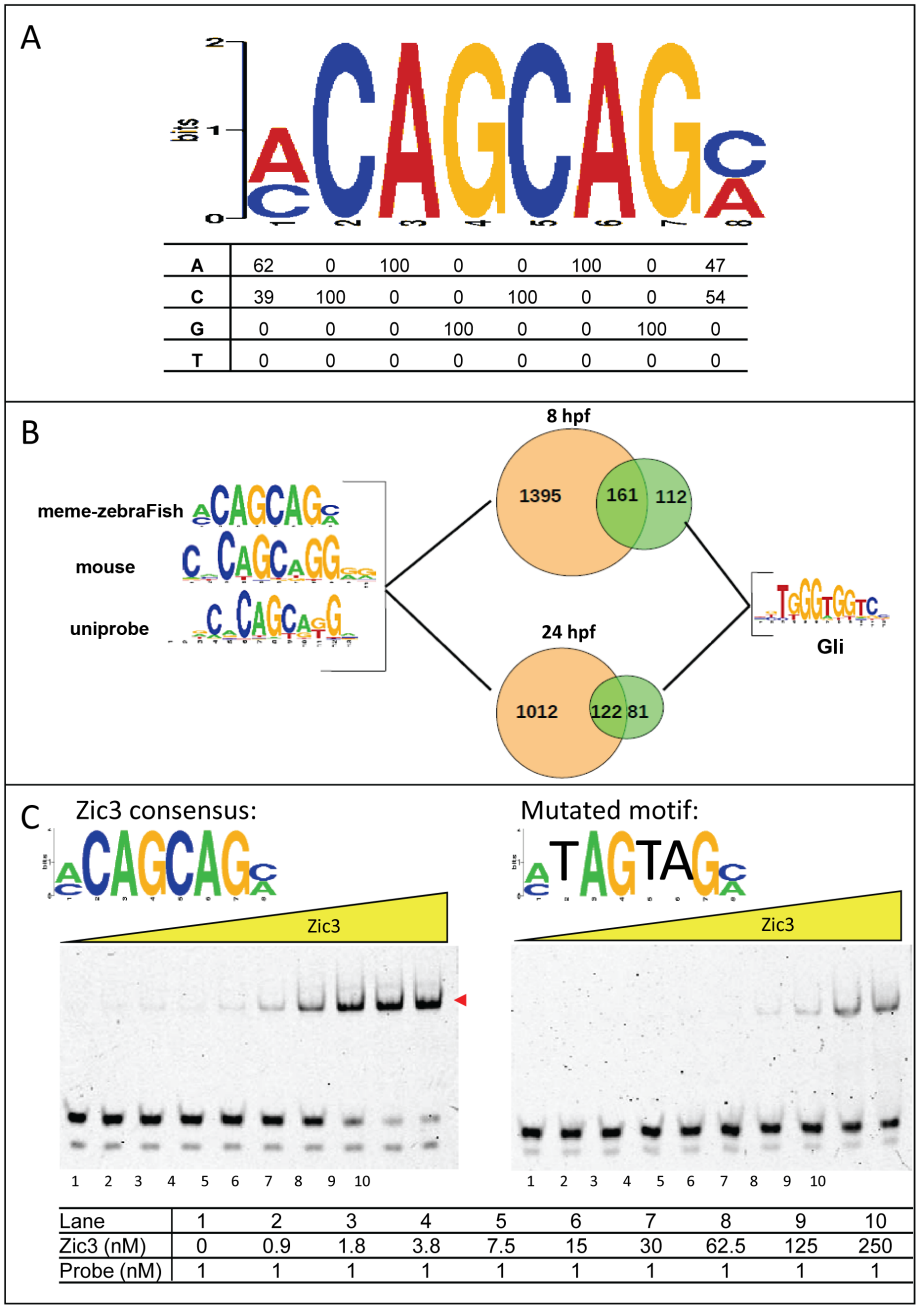
Zic3 binds specifically to its canonical motif. A, Zic3 consensus motif identified through interrogation of the top 1000 ChIP-seq peaks according to its statistical significance. B, pie-chart showing the frequency of occurrence of the most common binding motif and the Gli motif. C, EMSA with Zic3 canonical motif probe (left panel) and a mutated sequence probe (right panel) demonstrated the specificity of Zic3 binding to its consensus motif. Cy-5 labeled probes containing consensus and mutated motifs were incubated with increasing concentrations (yellow triangle) of Zic3 recombinant protein (Zic3_ZF2-5). A positive shift is indicated by a decrease in mobility (red arrowheads).

To identify the consensus motif in Zic3-binding sites, we performed *de novo* motif search using sequences within 50 bp (total length 100 bp) of the top 1000 peaks summit. The highest scoring motif in both datasets consisted of a CAGCAG core (Fig. 3A) and was similar to that previously identified in mouse ES cells using ChIP-chip [38] (Fig. S3A) and Zic3 motif in UniPROBE database [39]. This motif occurred in 48.5% (1556/3209) of 8 hpf peaks and 54.3% (1134/2088) of 24 hpf peaks (Fig. 3B). This consensus motif was bound in a dose-dependent manner by a recombinant protein encompassing the Zic3 DNA binding domain (Zic3_ZF2-5; Fig. 3C). This binding was reduced upon introducing three-point mutations to the motif, confirming binding specificity. The mouse Zic3 recombinant protein mZic3-DBD-HisMBP [38] also recognized the consensus motif derived from the zebrafish genome (Fig. S3B), demonstrating cross-species conservation of Zic3 consensus motif. On the other hand, two other motifs enriched in the dataset to a lesser extent were not specifically recognized by Zic3_ZF2-5 recombinant protein (Fig. S3C). Enrichment of these motifs among the identified peaks might signify an indirect binding of Zic3 to these sequences through interaction with other TFs. Interestingly, Gli motif was found in both 8 hpf and 24 hpf datasets (273 peaks, 8.5% in 8 hpf; 203 peaks, 9.7% in 24 hpf; Fig. 3B). More than half of peaks containing Gli motifs also had an adjacent consensus Zic3 motif at both developmental stages, in support of interactions between Gli and Zic3 [40], [41].

### Zic3 regulates target genes involved in early development

**Figure 4:**
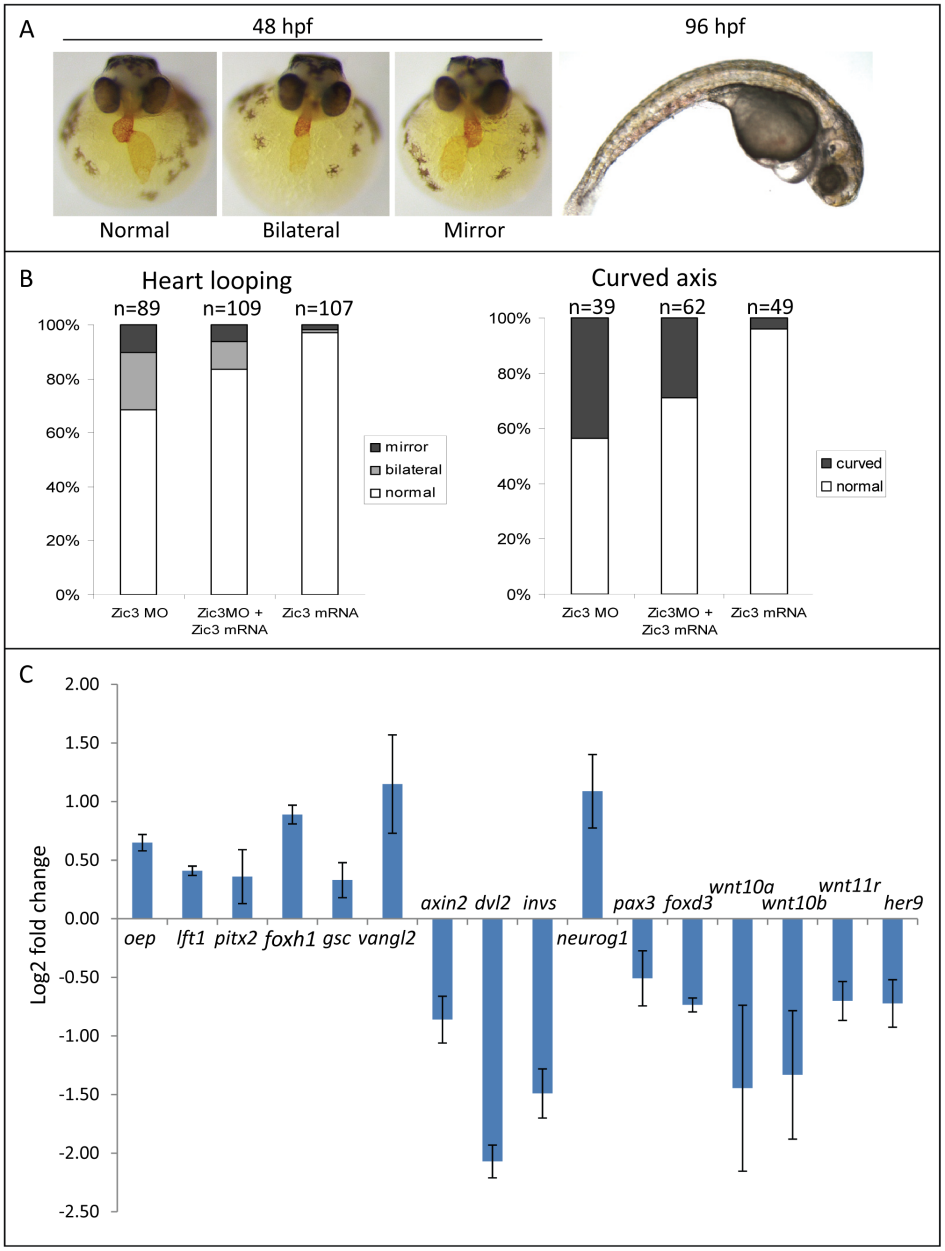
Zebrafish phenotype as a result of Zic3 knock-down. A, MF-20 antibody staining of heart, showing normal, bilateral, and reversal of looping at 48 hpf. Curvature of body axis and edema of the heart in Zic3 morphants at 96 hpf. B, percentage of embryos with heart laterality and axis curvature defects observed at 48 hpf in embryos injected with Zic3 MO alone, Zic3 MO and Zic3 mRNA, and Zic3 mRNA alone. C, expression levels of selected marker genes measured by qRT-PCR. Assay was performed in three independent groups of embryos injected with 3.4 ng of Zic3 morpholino. Marker genes *oep* through *invs* was assayed at 8 hpf, *neurog1* at 10 hpf, and *pax3* through *her9* at 24 hpf.

To identify Zic3 target genes during gastrulation and early neural development, we profiled the transcriptome of 8 hpf embryos after Zic3 morpholino (MO)-mediated knockdown. Embryos injected with the same MO dosage as in Cast *et al.* [10] exhibited similar gastrulation and convergent extension (C-E) defects (data not shown). However, to minimize the detection of non-direct targets in microarray, we injected the embryos with a lower dose of MO (1.7 ng in our experiments versus 7.5 ng in [10]) which did not cause visible morphological defects during gastrulation (refer to Methods section), but affected heart laterality and caused curvature of the A-P axis at later stages (Fig. 4A). These phenotypes were rescued by co-injection with Zic3 mRNA which, when injected alone, had little effect (Fig. 4B). This confirmed the specificity of the phenotypes caused by Zic3 MO injection.

**Figure 5:**
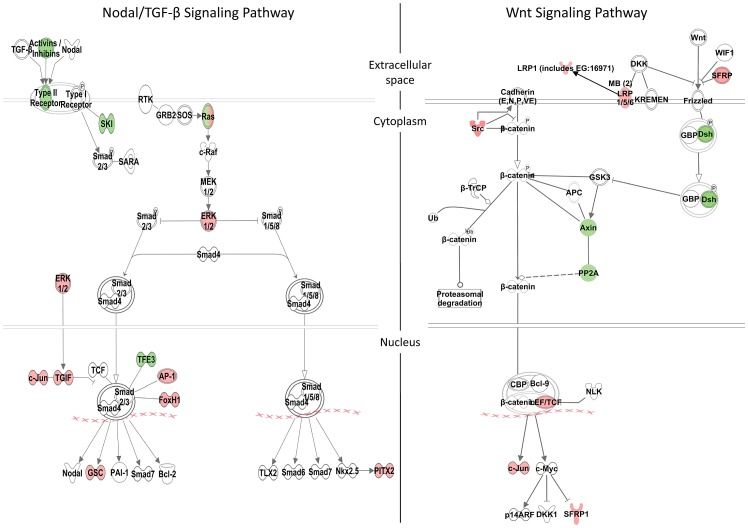
Zic3-regulated genes in the Nodal and Wnt signaling pathways. Schematic diagram of the Nodal/TGF-β and Wnt signaling pathways generated by the Ingenuity Pathway Analysis Software. Genes differentially regulated by Zic3 are shown in colour – red for upregulation and green for downregulation of their expression patterns in the microarray data.

We identified 1316 genes differentially expressed in MO injected embryos (morphants, fold change >1.2; p≤0.05; Table S3). GO analysis revealed prominent enrichment in functions related to embryonic morphogenesis (Table S4). When the same or higher dose of MO (3.4 ng) was injected, the expression of several representative genes showed similar trend when measured by qPCR. This validated a possibility of their regulation by Zic3 (Fig. 4C; Table S7). We then determined the presence of Zic3 binding peaks within 100 kb of the TSS of these differentially expressed genes, which we defined as a selection criterion for Zic3 target gene. Based on this selection, 454 genes out of the total 1316 were identified as putative targets of Zic3 (Table S5 and Table S6). This set contains genes of the Nodal signaling pathway such as *oep*, *lft1* and *pitx2* (Fig. 5). While the presence of Zic3 binding in association with *oep* suggests direct regulation of Nodal pathway, the association of Zic3 peaks with *lft1* and *pitx2* suggests that Zic3 could also regulate the pathway through its modulators [42], [43]. These three genes, along with other members of this pathway not associated with Zic3 peaks (*foxh1*, *bon*, and *gsc*), were concurrently upregulated in Zic3 morphants (Fig. 4C; Table S3) suggesting negative regulation of the Nodal pathway by Zic3. Inhibition of Nodal signaling indicates suppression of endodermal fate [15], [44]–[46]. This correlated with broader expression of endodermal marker *sox17a* in 8 hpf Zic3 morphants (Fig. S4A). The inhibition of endodermal development by Zic3 is in line with previous observation in murine ES cells [38]. Similarly, peaks were associated with three genes of the canonical Wnt signaling pathway: *axin1*, *jun*, and *vent* (Table S5). In support of this association, microarray analysis revealed that the negative regulator of canonical Wnt pathway *axin1* was downregulated in Zic3 morphants, while the downstream components *jun* and *vent* were upregulated (Fig. 5; Table S3). The expression of some other members of this pathway (*axin2* and *nlk1*) without association with peaks has changed in Zic3 morphants based on microarray data. This implied that such genes could be the indirect targets of Zic3. Such observation provided further support for Zic3 regulation of the canonical Wnt pathway. The inhibition of canonical Wnt signaling by Zic3 was previously reported in frogs as a mechanism for organizer development [16]. Interestingly, Zic3 LOF only affected downstream components of these signaling pathways, and not the ligands, suggesting that at 8 hpf Zic3 is more likely to modulate the response to Wnt signaling in the target cells rather than initiation of signaling.

Apart from genes previously implicated as targets of Zic3, the combined ChIP-seq and microarray screen also identified novel candidates. Zic3 peaks were found in association with genes known to regulate cell proliferation in the neural plate, *dlx4b* and *msxe* [47], [48]. These genes perform a function [49], [50] similar to that of *msxc*, *irx1a*, and *irx7*, which do not have associated peaks but were nevertheless downregulated in Zic3 morphant (Table S3; S7). This observation suggests the role of Zic3 in promoting proliferation of neural progenitors at 8 hpf. Since these genes are known to inhibit neural differentiation, we assayed the expression of proneural gene *neurog1* [51] in Zic3 morphants at 10 hpf. As expected, *neurog1* was upregulated, in concert with the downregulation of *her9* (Fig. 4C; Table S7), which provided further support for Zic3 role as a promoter of proliferation of neural progenitors and repressor of neural differentiation.

More interestingly, the novel candidate targets include members of the non-canonical Wnt signaling pathway (*dvl2*, *rock2b* and *invs*). These genes were co-expressed with *zic3* during gastrulation (Fig. S5A, B) and were downregulated in the microarray (Table S3; Fig. 4C). One of the non-canonical Wnt pathways, the planar cell polarity (PCP), regulates convergence-extension (C-E) [52] and controls the positioning of the motile cilia [53]. The changes in expression of *sox17*, *ntl*, *pax3a* and *sox19a* mark correspondingly, endoderm, mesoderm, neural crest and neural plate. The broadening of their expression domains suggested that in Zic3 morphants C-E is affected (Fig. S4B–D, [10]). On the other hand, the disorganized expression of *foxj1a* and *sox17a* in the dorsal forerunner cells at an earlier stage indicated abnormalities of their migration in Zic3 morphants (Fig. S6), which may lead to abnormalities in L-R patterning. A correlation between C-E defects and L-R defects in Zic3 morphant was reported [14], suggesting Zic3 regulation of these events through the non-canonical Wnt pathway.

Several genes implicated in cell migration and polarity were among the targets. These include *npy* [54], *ptenb* [55], *sepn1*, *srsf1a* [56], and *sparc* [57], [58], all of which were downregulated in microarray and associated with peaks. WISH analysis showed that their expression overlap that of *zic3* (Fig. S5C; ZFIN; University of Oregon, Eugene, OR 97403-5274; URL: http://zfin.org/; 21 June 2013). In addition, other genes with similar function, such as *ccdc88a* (probe generated from BC057440 which correspond to the annotated *ccdc88a* sequence) [59], [60] and *tsg101* [58], were also downregulated in the microarray despite not having associated peaks. Hence the direct and indirect regulation of these genes by Zic3 could be the mechanism behind cell movements during gastrulation.

### Zic3 regulates a distinct set of target genes and developmental processes at 24 hpf

**Figure 6:**
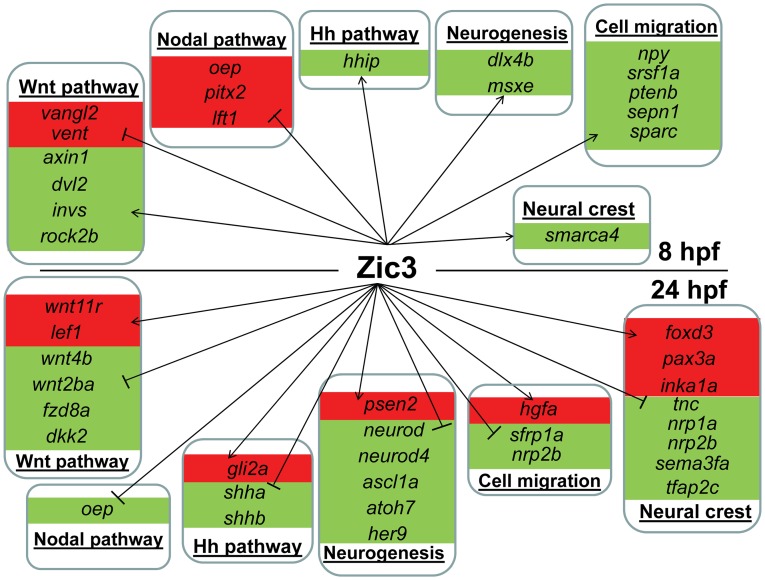
Candidate target genes regulated by Zic3 at 8 hpf and 24 hpf developmental stages. Target genes are grouped based on their signaling pathway or functions. Changes in expression in microarray are represented by red and green backgrounds for up- and down-regulation respectively.

To identify potential *zic3* targets during late neurogenesis, we performed microarray expression analysis on 24 hpf GFP-positive *zic3* expressing cells that were FACS-sorted (Table S8). Comparing expression levels to a control dataset derived from GFP-negative cells (cells negative for *zic3* expression), we identified genes enriched in GFP-positive cells (*zic3*-expressing cells). A total of 689 genes (p-value<0.05; fold change ≥1.5) were enriched in *zic3*-expressing cells (*zic3*-coexpressed genes). Among these genes were six members of the Zic family and other genes expressed in the dorsal neural tube. This confirmed the identity of the sorted cells as dorsal neural cells. Among the *zic3*-coexpressed genes, 167 had at least one peak within 100 kb of their TSS, rendering them putative Zic3 targets (Table S10). Similar to the 8 hpf stage, members of the Wnt pathway were also among the targets. However, Zic3 seems to regulate a different set of Wnt components, including *wnt11r* and *lef1* (Fig. 6, Table S8). qRT-PCR revealed that *wnt11r*, were down-regulated in Zic3 morphants at 24 hpf (Fig. 4C; Table S7), confirming their positive regulation by Zic3. Two other genes encoding Wnt ligands, *wnt10a* and *wnt10b*, were co-expressed with *zic3*, and regulated upon Zic3 knockdown (Table S7; Fig. 4C) although they were not associated with peaks in ChIP-seq, suggesting that they may be indirect targets of Zic3. A striking difference between 8 hpf and 24 hpf regulatory landscape is apparent from the distinct functions associated with Zic3 target genes at each stage. For example, many genes regulating cell migration and polarity were identified as Zic3 targets at 8 hpf, whereas at 24 hpf neural crest determinants were found. The latter included *foxd3*, and *pax3a* which were further confirmed to be responsive to Zic3 knockdown (Fig. 4C, Table S7, S11).

On the other hand, in *zic3*-negative cells, 835 genes were enriched by at least 2-fold (non *zic3*-coexpressed genes enriched for endoderm and mesoderm-specific expression terms, Table S9). Among these, 195 had peaks within 100 kb of their TSS, suggesting repression of these genes in cells expressing *zic3* (Table S10). Several proneural genes (*neurod, neurod4, ascl1a*) were found under this category, which may reflect that the *zic3*-expressing cells in the dorsal neural tube are not differentiating. Interestingly, the presence of a Zic3 peak in association with *oep* suggests that a similar inhibition of Nodal by Zic3 occurs at both 8 hpf and 24 hpf (Fig. 6).

Taken together, an entirely different set of candidate Zic3 target genes were found at 24 hpf compared to 8 hpf (Fig. 6). Although similar signaling pathways, such as the Wnt and Nodal pathways, were regulated by Zic3 at both developmental stages, different members of these pathways were targeted by this regulation at each stage. Furthermore, the global shift in Zic3 binding sites from 8 hpf to 24 hpf suggested the presence of complex regulatory changes accompanying developmental transitions.

### Zic3 preferentially binds to distal regulatory elements and regulates developmental genes

The large number of Zic3 binding sites in the distant intergenic regions suggested that Zic3 may direct the expression of target genes by binding to the distal regulatory elements. In support of this idea, relevant biological categories could be observed among genes associated with peaks located outside of their basal regions of −5 kb to +1 kb of TSS (2716 genes; Table S2; Fig. S7A) or at a distance more than 50 kb (989 genes; Table S2; Fig. S7B). In contrast, no particular enrichment of GO categories could be observed for 119 genes associated with peaks in their basal region (Table S2). Of these, 77 had expression data in ZFIN (University of Oregon, Eugene, OR 97403-5274; URL: http://zfin.org/; 21 June 2013), but none of these were co-expressed with *zic3* at 8 hpf, while only 6 (*lppr3a, p2rx3b, lingo1b, myo15aa, robo4, gng3*) had expression overlapping with *zic3* at 24 hpf (not shown).

**Figure 7:**
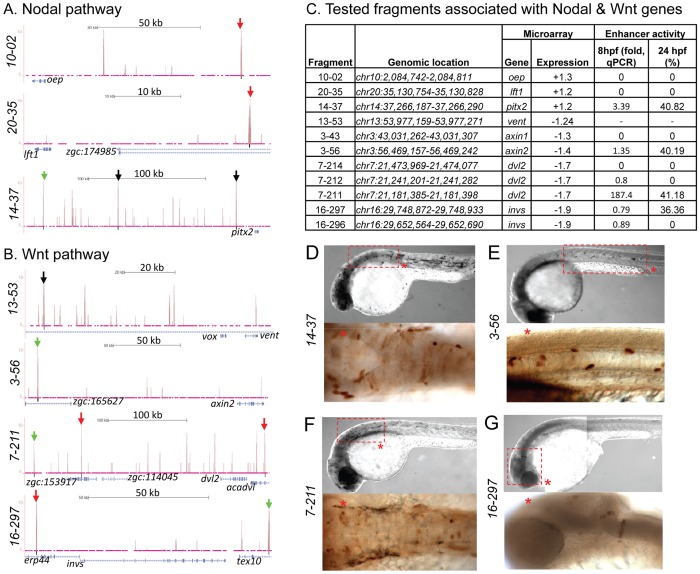
Zic3 binding sites associated with genes from the Nodal and Wnt pathway genes. UCSC browser image depicting genomic locations of Zic3 peaks identified near Nodal (A) and Wnt (B) pathway genes at 8 hpf. Single black vertical bars below histogram - peaks called by QuEST algorithm, blue horizontal bars - annotated exons (tall boxes), UTRs (half-sized boxes), introns (lines, arrowheads denote transcript orientation); Zic3 binding sites with negative (red arrows) and positive (green arrows) enhancer activity. Scale bars are indicated by black horizontal line at the top of each panel. C, list of tested fragments associated with Nodal and Wnt pathway genes. Enhancer-driven expression was assayed by qRT-PCR of *gfp* at 8 hpf and through microscopic observation of GFP expression pattern at 24 hpf. Between 50 to 100 embryos were assayed in each experimental time point. D–G, representative figure of *gfp* expression driven by selected fragments of Zic3 binding sites in F_0_ embryos at 24 hpf, immunostained with anti-GFP antibody. D*, F*, dorsal view; E*, G*, lateral view.

To test whether peaks associated with distal genes function as regulatory elements, we used the enhancer activity reporter assay [61]. We chose five distal peaks associated with genes from Nodal and Wnt signaling pathways, including *oep* (fragment 10-02, 94.7 kb downstream from TSS), *axin1* (fragment 3-43, 71.53 kb downstream), *lft1* (fragment 20-35, 29.77 kb downstream), *dvl2* (fragment 7-214, 55.92 kb downstream), and *invs* (fragment 16-297, 78.08 kb downstream). A canonical Zic3 motif was present within 100 bp of each peak summit except for fragment 10-02. Only fragment 16-297, associated with *invs*, showed enhancer activity (Fig. 7B,C,G; Table S12). When the association region was extended to 500 kb, we found more peaks associated with *dvl2* (fragment 7-211, 236.6 kb upstream), *axin2* (fragment 3-56, 147.9 kb upstream), and *pitx2* (fragment 14-37, 180.32 kb upstream). These peaks had at least one canonical Zic3 motif and exhibited positive enhancer activity (Fig. 7, Table S5, S12). Intriguingly, some of the expression patterns driven by the tested enhancers only partially matched that of the associated genes (fragments 14-37 and 3-56; Fig. 7D,E), which could be due to functional dependence on interaction of multiple regulatory elements [62], [63]. Nevertheless, the presence of Zic3-binding sites with an enhancer activity near genes responding to Zic3 LOF suggested that these genes were direct targets of Zic3.

To validate the activation of the enhancer fragments by Zic3, we co-injected fragment 7-211, which drove the strongest reporter gene expression at 8 hpf and 24 hpf (Fig. 7C), and Zic3 MO into the zebrafish embryo. When assayed by qRT-PCR at 8 hpf, a significant decrease in reporter expression in a MO dose-dependent matter was observed (Fig. S8). No reduction in reporter expression was observed when control MO was used. A similar result was obtained when two other fragments, 4-16 and 17-24 which coincided with CNEs (Tables 1, S12), were tested (Fig. S8), demonstrating Zic3-dependent induction of reporter expression through these fragments.

**Table 1:** List of CNE and non-CNE fragments tested using *in vivo* enhancer assay.

name	Location			Flanking genes (within 100 kb)	Enhancer activity	
	chr	start	end		8 hpf (fold, qPCR)	24 hpf (%)
**CNEs**						
13-29	chr13	29550477	29550578	*zgc:153142, unc5b, chat*	1.88	29.69
17-24	chr17	24095622	24095712	*otx1b*	1.42	34.48
15-26	chr15	26812885	26812985	*brip, tbx4, tbx2b*	2.07	42.31
7-28	chr7	28533221	28533276	*sox6*	1.16	21.74
4-16	chr4	16204706	16204764	*sox5, casc1, bcat1*	0.91	0
13-16	chr13	16987933	16987979	*c10orf11*	1.66	45.16
20-4	chr20	4566963	4567026	*-*	1.1	0
23-29	chr23	29550522	29550546	*tardbp1, sst6, pgd, kif1b*	1.87	16.28
8-34	chr8	34893735	34893847	*pbx3b*	2.62	53.42
21-14	chr21	14240602	14240635	*zgc:101080, zgc:183801, lhx5*	4.99	0
1-22	chr1	22884044	22884087	*mab21l2*	1.55	0
17-33	chr17	33138334	33138419	*prox1, smyd2a*	1.41	68
9-32	chr9	32394294	32394354	*zic5, zic2a*	2.04	7.69
25-15	chr25	15101126	15101143	*dnajc24, mpped2*	1.16	6.56
19-42	chr19	42355789	42355849	*shfm1, dlx6a, dlx5a*	1.26	0
**Non-CNEs**						
12-6	chr12	6034509	6034630	*dlx4b, zgc:163073, ghrhr2, dlx3b*	1.89	0
16-296	chr16	29652564	29652690	*invs, tex10, erp44*	0.79	0
17-245	chr17	24570163	24570239	*fam54b, sepn1, spdya*		0
16-34	chr16	34391029	34391168	*prpf31, leng1, cnot3a, mboat7, zgc:92763*	3.01	0
20-33	chr20	33776501	33776557	*hen1, fam102bb, zgc:110463, rock2b*	1.06	
17-25	chr17	25263319	25263482	*zgc:165525, srrm1, clic4, zgc:154055, lck*	0.89	0
18-19	chr18	19527760	19527861	*sma3b, aagab, iqch, LOC564395*	0.71	0
6-28	chr6	28633320	28633360	*tp63, tomm70a, tbc1d23, glmnb, gfi1.2*	1.44	0
23-7	chr23	7756857	7756924	*pofut1, kif3b, plag12*		0
14-09	chr14	95269	95403	*zgc:158483, otop1, nkx3.2, zgc:110421,*	1.17	0
7-212	chr7	21241201	21241282	*zgc:153917, zgc:114045, zgc:64136, prox1b*	0.8	0
3-42	chr3	42787879	42787947	*litaf, snn, zc3h7a, zgc:92162*	1.49	0

Enhancer activity at 8 hpf was assayed by qRT-PCR of *gfp* transcript. Enhancer activity at 24 hpf was determined through observation of GFP expression by immunohistochemistry staining. A positive enhancer activity is defined as either a positive enrichment (at least 1.5 fold by qRT-PCR) of *gfp* expression at 8 hpf or a consistent expression pattern other than the background *cFos* expression (in the muscle and blood cells) in at least 10% of injected embryos at 24 hpf. Typically, 50 to 100 injected embryos were assayed in each experimental time point.

### Conserved Zic3 binding sites act as enhancers

**Figure 8:**
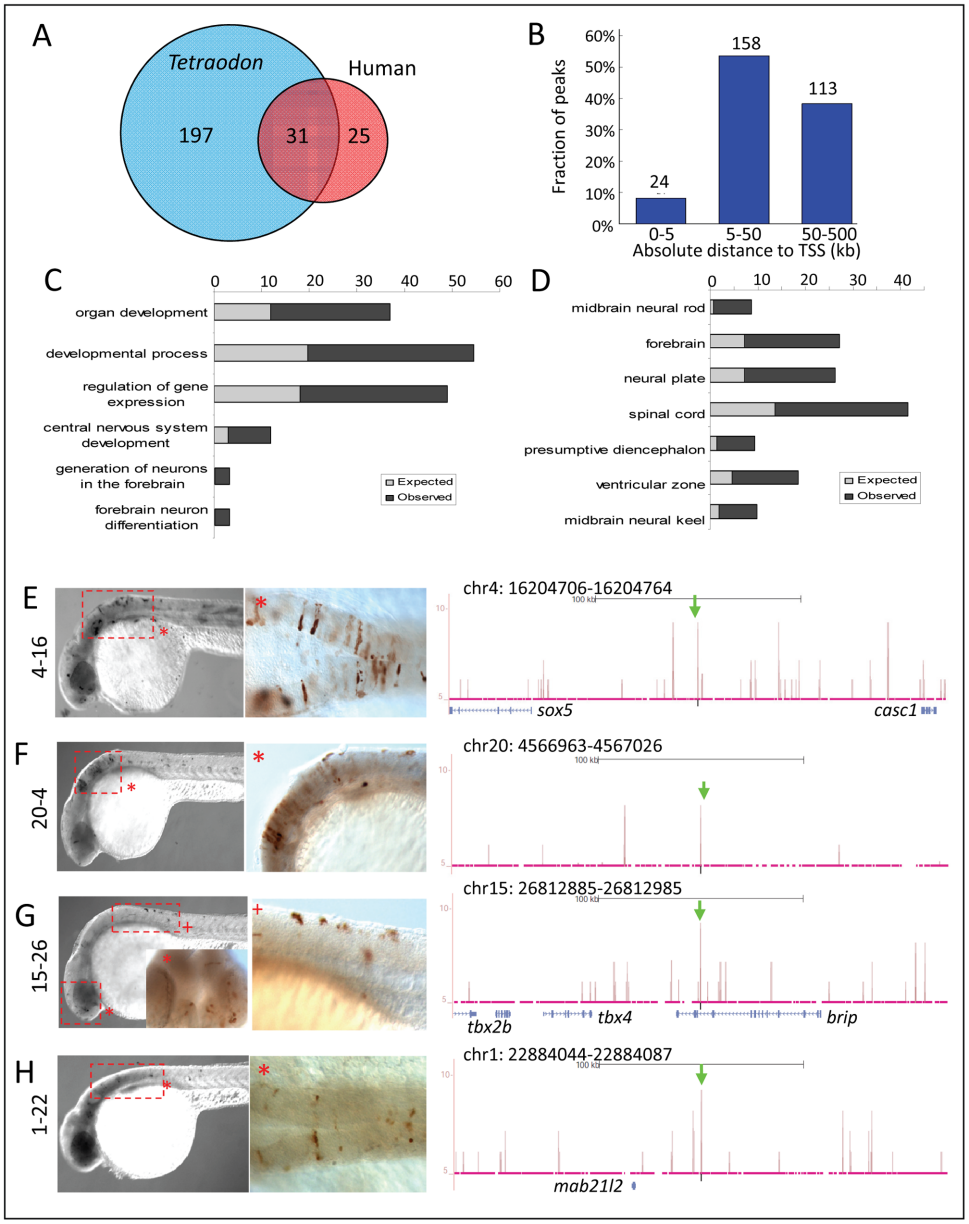
Zic3 regulates neural-specific expression through CNEs. A, number of CNEs found among the Zic3 peaks. A large subset was conserved between zebrafish and *Tetraodon*, while a smaller subset was conserved between zebrafish and human. B, distribution of CNE peaks with regards to their distance from TSS of genes. Enrichment of biological process (C) and tissue-specific expression (D) terms among genes associated with CNE peaks. Light and dark grey bars represent expected and observed enrichments of functional categories according to DAVID GO terms. E–H, representative figure of 24 hpf F_0_ embryos expressing *gfp* (left panel) driven by Zic3 CNE peaks shown in UCSC browser image (right panel, green arrows); black horizontal line at the top of each panel represents 100 kb. E*, H*, dorsal view; F*, G*, lateral view.

To study whether Zic3 binding sites were evolutionarily conserved, we overlapped the 8 hpf dataset with a list of known conserved non-coding elements (CNEs; ANCORA database) [64].We identified 228 peaks as CNEs conserved between zebrafish and *Tetraodon*, and 56 as CNEs conserved between zebrafish and humans (Fig. 8A), with 31 in common between the two groups. Similar to the distribution profile of the full set of peaks, these CNE peaks were mostly located outside of the basal promoter region (Fig. 8B). Genes associated with these CNEs were enriched for developmental functions and neural tissue-specific expression (Fig. 8C,D; Table S2).

Of 15 CNE peaks tested for enhancer activity, 11 (73%) drove *gfp* expression at either 8 hpf or 24 hpf, or both (Table 1). Of these eleven, eight drove higher *gfp* expression compared to the reporter vector alone at 8 hpf (fold change at least 1.5 compared to enhancer-less vector). Of these eight, four continuously drove reproducible tissue-specific *gfp* expression in various regions of the CNS up to 24 hpf (Fig. 8E–H), which overlapped with known expression domains of *zic3* (Fig. 1F). Another three CNE peaks drove reporter expression only at 24 hpf. The CNE peaks with enhancer activity included the fragments 4-16 and 20-4, which drove expression in the brain, eye and trunk. In the hindbrain, both drove similar expression in neuroepithelial cells with radial morphology. In the trunk, activity of 4-16 was detected in muscle cells, whereas that of 20-4 was largely confined to the neural tube (Fig. 8E,F). The *gfp* expression pattern driven by 4-16 partially recapitulated that of a nearby gene, *sox5*. On the other hand, 20-4 was located in a gene desert region, suggesting long distance regulation. Fragment 15-26 drove *gfp* expression largely in cells along the neural tube (Fig. 8G), which partially recapitulated the expression of *tbx2b* nearby. Fragment 1-22 drove *gfp* expression mainly in the hindbrain region (Fig. 8H), which partially recapitulated that of the nearby *mab21l2*.

On the other hand, out of 12 non-CNE peaks tested only two (17%) drove higher *gfp* expression than the reporter vector alone at 8 hpf (Table 1). Together with the fragments corresponding to peaks associated with microarray-identified genes, out of 35 fragments tested for activity as enhancers, 17 (49%) were positive. Two thirds of the active peaks were previously identified as CNEs. Whereas this indicated somewhat better chance of finding enhancers amongst CNEs, it also suggested that a significant number of enhancers are not conserved in evolution.

## Discussion

The majority of Zic3 binding sites were found outside promoter regions. While this could be partially attributed to the incomplete annotation of promoter regions in the zebrafish genome, the predominantly distal distribution of Zic3-binding sites revealed that Zic3 regulates transcription largely via distal regulatory elements. Such distribution of binding sites was previously observed in other genome-wide analyses of several TFs in cell culture or mammalian tissues [21], [22], [25], [65]. Our findings therefore establish that a similar distal regulatory mechanism is in effect within the context of Zic3 function during development *in vivo*.

Some of the Zic3 binding sites overlapped with CNEs, most of which drove expression in neural tissues. CNEs are known to regulate developmental genes [66]–[69]. However, in our dataset CNEs represented only 5% of the total Zic3 binding sites identified, while the majority was under weak evolutionary constraint. Tissue-specific enhancers have been shown to differ in the extent of evolutionary conservation of their sequence [70], [71]. Having only 5% overlap with CNEs, the set of Zic3-binding sites showed a similar trend. The lack of sequence conservation could be explained by the relaxation of selection pressure towards regulatory elements [72] owing to the genome duplication event in teleosts [73]–[75]. Given that at least for now the data available in zebrafish and mammals suggest that only a minority of sites are conserved in both classes of animals, other explanations should be considered. Detailed characterizations of other TFs in the zebrafish would provide a better understanding of the extent of conservation in regulatory regions in teleosts.

Cell culture studies have demonstrated interactions between multiple enhancer elements in regulating the transcription of a target gene [24], [62], [63], [76], [77], as well as interactions between a TF and different binding partners which can result in alternative transcriptional outputs [26], [78], [79]. Our results provide an insight of such complexity of transcriptional regulation by Zic3 in developmental context *in vivo*. For instance, the concurrent upregulation and downregulation of different subsets of direct target genes by Zic3 suggest that Zic3 binding can result in either activation or repression of target genes, and implies that additional mechanisms determine these two outcomes. Another facet of the data revealed distinct Zic3 binding profiles at 8 hpf and 24 hpf. The genes associated with binding events at these two stages showed relevant functional enrichments. This shift in binding was not dictated by a change in DNA recognition motif as almost identical dominant motifs were identified in both stages.

The combinatorial analysis of ChIP-seq and microarray datasets revealed an entirely distinct set of candidate Zic3 target genes at 8 hpf and 24 hpf. Whereas not totally unexpected, this analysis revealed some surprises. First, a developmental switch towards regulation of different members within the same signaling pathway was detected. In the context of Wnt signaling this shifted Zic3 impact from the intracellular part of Wnt signaling towards extracellular ligands in this pathway. Second, that cells expressing Zic3 show a reduced level of transcription of proneural genes placed an impact of Zic3 on cells that are in a state either before or after neural differentiation. Zic3 has been linked with pluripotency of stem cells in mammals [80]. Whereas it is less likely that Zic3 positively regulates the proneural genes at 24 hpf, at the same time this does not exclude a possibility that it could be involved in this process (as suggested [2]) during earlier stages. Taken together, these observations suggest that functional relationship between Zic3 and its target gene could not be deduced from a simple one-to-one interaction model. Factors, such as the presence of different subsets of interacting partners or accessibility of certain binding sites as dictated by chromatin states, in different spatiotemporal contexts may affect transcriptional output.

One implication of an interactive regulatory landscape is that genes targeted by a particular TF may not be determined by simply observing binding of the TF near its genomic locus. Additional proof, such as responsiveness of the particular target gene to LOF of the TF, would be necessary. In our data, there is a surplus of Zic3 binding events compared to those associated with responsive target genes. Widespread binding of TFs exceeding their known target genes have been reported in cell culture and in *Drosophila* [81]–[87] and is suggestive of non-functional binding. This may happen due to interaction of TFs with randomly occurring target sequences in the genome [78], [88]. The availability of expression data helps to identify candidate target genes within the vicinity of a TF binding event by providing additional functional cues. Nevertheless, given that TF-target genes interactions could occur over long distances [22], [89], [90], it is still possible that seemingly isolated Zic3 binding events with no responsive genes within a set distance criteria might actually be regulating a target located further away. Until a more detailed understanding of the architecture of genome-wide interactions have been achieved, this possibility could not be ruled out.

The highly interconnected TF regulatory network also necessitates a careful interpretation of enhancer function by reporter assays: while such assays can be useful to identify independently acting regulatory elements, evidence exists for regulatory elements acting in tandem, resulting in higher transcriptional output [24], [62], [63], [76], [77]. While other possibilities such as non-functional occupancy and repressive interactions could not be ruled out, the TF interaction model could account for the inactivity of several of the tested enhancers inferred from the reporter assay. The occurrence of Zic3 consensus motifs in close proximity to 50% of peaks containing Gli consensus motif supports this idea. Interestingly, the presence of Gli motifs does not seem to be specific to a particular developmental stage, as both 8 hpf and 24 hpf data show similar proportions of Zic3 peaks containing Gli motifs nearby. As *in vitro* data have demonstrated physical and functional interactions between Zic and Gli proteins [40], [41], such interaction, as well as interactions with other binding partners, may also occur *in vivo* in regulating transcription of target genes.

Our identification of novel target genes of Zic3 has improved an understanding of the mechanism by which Zic3 regulates development. These results demonstrated that Zic3 inhibits Nodal signaling (either directly or indirectly) which is implicated in mesendodermal specification [15], [44]–[46]. Similarly, Lim and colleagues [38] observed that murine ES cells acquired endodermal fate upon Zic3 knockdown, which supported an idea that Zic3 acts as an inhibitor of endodermal fate. Coincidentally, Nodal and Wnt signaling is known to regulate gastrulation [91]–[94]. Their regulation by Zic3 therefore may account for the gastrulation defect observed in Zic3 morphants. On the other hand, proper midline development during gastrulation is essential for proper L-R patterning [15], [95], [96]. Therefore, an involvement of Zic3 in regulating gastrulation through Nodal and canonical Wnt *per se* could have been sufficient to ensure a proper L-R asymmetry. However, our results suggested that Zic3 may also regulate the non-canonical Wnt (PCP) signaling pathway which is implicated in ciliogenesis. Interaction of these signaling pathways culminates in the establishment of a proper embryonic L-R axis [97]–[102]. Therefore, we could not rule out the possibility of direct involvement of Zic3 in later events specific to L-R patterning. In this context, it is noteworthy that *mkks* was also found as one of the Zic3 targets (Table S5) which is implicated in both L-R patterning and C-E movements during gastrulation through interaction with *vangl2* [103]–[106]. Therefore, the regulation of non-canonical Wnt signaling by Zic3 could be at a core of developmental events linking C-E movement and L-R patterning [10].

Our finding that Zic3 regulates genes implicated in proliferation of neural progenitors agrees with the idea that Zic3 has properties of a stem cell factor [38], [80]. A mode of Zic3 regulation of genes responsible for the proliferation of neural progenitors reconciles the role of Zic3 in both early neuroectodermal specification and later events of neurogenesis. In essence, it establishes a particular role of Zic3 (and possibly other Zic family members) as an important regulator of proliferation of neural progenitors [7]. This model challenges previous assumptions that Zic3 induces the expression of proneural genes shown in overexpression studies [18], and suggests that an activation of proneural genes could be a downstream consequence of Zic3 regulation of proliferation of neural progenitor at an earlier stage of neurodevelopment. Given that *neurog1* expression was upregulated upon Zic3 knockdown, and Zic3 binding sites were found near *neurog1*, as well as other proneural genes such as *neurod4* and *ncam1a*, Zic3 may have an additional direct role in neural differentiation as its inhibitor. This possibility is also supported by the downregulation of *her9*. This places Zic3 within a regulatory landscape of Notch signaling in support of an early hypothesis based on functional analysis of Zic1 [107].

## Materials and Methods

### Zebrafish

Zebrafish of wild type (AB strain) and transgenic line SqET33 [28], [34] were maintained according to established protocols [108] following all the ethical practice recommended for fish maintenance. Embryos were staged according to standard morphological criteria [109].

### Fluorescence-Activated Cell Sorting (FACS)

Dechorionated 24 hpf transgenic embryos were deyolked in PBS by pipetting through the 1 ml pipette tip. Cells were dissociated with trypsin solution (0.05% trypsin and 0.2 mM EDTA) in PBS for 15 min at room temperature. To facilitate dissociation of cells, embryos were pipetted through the 200 µl pipette tip. Trypsin was inhibited with complete protease inhibitor cocktail (Roche) and cell suspension was filtered through a nylon mesh (40 µm Cell Strainer, BD Falcon). Immediately, an equal volume of 4% paraformaldehyde (PFA) in PBS was added to cell suspension and cells were fixed for 10 min at room temperature. Reaction was stopped by an equal volume of ice-cold 0.25 M glycine in PBS, cells were washed three times with 0.125 M glycine-PBS and resuspended in the same buffer. Cell sorting was carried out with FACSAriaII Cell Sorter (BD Bioscience). To set autofluorescence level, cell sorter was calibrated with PFA-fixed GFP-negative cells before cell separation. GFP-positive and GFP-negative cells were collected in 0.125 M glycine-PBS, frozen in liquid nitrogen and kept at −80°C until use. For microarray analysis, PFA fixation step was omitted and cells were sorted into complete L-15 Leibovitz medium (Gibco) containing 20% fetal bovine serum.

### ChIP-seq

Chromatin Immunoprecipitation (ChIP) was performed according to an established protocol (Wardle et al., 2006) with an addition of a deyolking step according to Link and colleagues (2006), with modifications (see Text S1). ChIP DNA was sequenced on the Illumina Genome Analyzer II (Illumina, USA). Detailed ChIP-seq methods are described in Supplementary information. Sequencing reads were mapped to the zebrafish Refseq genome assembly (Zv9), following which peak finding was performed using the QuEST algorithm [110] using the following parameters: bandwidth = 30 bp, region size = 600 bp, and FDR q-value<0.01. Peaks mapped to unassembled chromosomal contigs, centromeric regions, telomeric regions, segmental duplications and peaks consisting of >70% repeat sequence were removed. The ChIP-seq data have been deposited in the Gene Expression Omnibus database under the accession number GSE41458. To validate the ChIP-seq performance, we carried out quantitative PCR (qPCR) on randomly selected peaks, 5 within promoter region and 16 at regions outside of gene promoters. Taking a fold-change of 2 as a cutoff for positive enrichment, the qPCR analysis validated all but one peak tested (Table S1).The Database for Annotation, Visualization, and Integrated Discovery (DAVID) [111], [112] and Genomic Regions Enrichment of Annotations Tool (GREAT) [35] was used to find gene ontology-enriched terms. Overlapping of 8 hpf and 24 hpf ChIP-seq signals around peaks was performed within a region of +/−2 kb from each peak summit. Notice that some peak regions in 8 hpf dataset were not detected as peaks in 24 hpf dataset but they could be having sufficient amount of ChIP-seq tags at 24 hpf because of true binding by Zic3. Similarly there were regions detected as peaks in 24hpf samples and not detected in 8hpf but they may be bound by Zic3 in both samples and be having enriched ChIP-seq tag count in both. Hence ChIP-seq signal based clustering further clarified the status of detected peaks. Motif search was performed with MEME *de novo* motif finder [113]. From the top 1000 peaks by statistical significance, we extracted sequences comprising +/−50 bp from the summit of each peak. After finding the similarity of de novo motif from MEME with other published Zic3 motifs [39], [80], the quantification of occurrence of these motifs was done on all ChIP-seq peaks. For this the sequences within 400 bp from the peak summit were matched with PWM of motifs and the best matching score were calculated. After having the best matching score a threshold was used to determine the presence of motif. The PWM-matching threshold value for each motif was calculated using simulation such that when 10000 sequences were randomly designed to have probability similar to corresponding nucleotides in its PWM then 85% of those sequences could be detected. CNE peaks were identified by comparing the 8 hpf ChIP-seq dataset against a list of known CNEs in ANCORA database [64]. We performed the comparison to both human and *Tetraodon* CNE database to take into consideration the genome duplication event during teleosts evolution, which relaxed selection pressure on the conservation of important developmental enhancers [68], [72].The genomic coordinates of each peak summit were extended by 500 bp on each side and compared against the genomic coordinates of CNEs identified through comparison with either human hg19 or *Tetraodon* tetNig2 assemblies. A threshold of at least 70% sequence conservation within every 50 bp was used to define CNEs in each species.

### Recombinant protein expression and EMSA

Two recombinant constructs of the zebrafish Zic3 protein were produced, the full-length protein (Zic3_ORF) and the DNA-binding domain encompassing Zn-fingers 2 to 5 (Zic3_ZF2-5, amino acid residues 273–391). DNA sequences corresponding to each domains were PCR-amplified using the following primers: Zic3_ORF: 5′-GGG GAC AAG TTT GTA CAA AAA AGC AGG CTT CGA AAA CCT GTA TTT TCA GGG CAG CTT ACG TGA AAT TGC G CTC-3′ and 5′-GGG GAC CAC TTT GTA CAA GAA AGC TGG GTT TAC TCC ACC TGA AAA CGG ACT TG-3′; Zic3_ZF2-5: 5′-GGG GAC AAG TTT GTA CAA AAA AGC AGG CTT CGA AAA CCT GTA TTT TCA GGG CGC CTT CTT CAG ATA CAT GCG-3′ and 5′-GGG GAC CAC TTT GTA CAA GAA AGC TGG GTT TAT GAT TCG TGT ACC TTC ATA TG-3′. Each forward and reverse primer contained an attB recombination site overhang, with an additional Tobacco Etch Virus (TEV) protease cleavage site in the forward primer preceding the N-terminal Zic3 coding sequence. Protein expression and purification was performed as previously described (Lim et al., 2010). Electrophoretic mobility shift assay (EMSA) was performed as previously described [38]. Briefly, Cy5-labeled oligonucleotide pairs (1^st^ BASE, Singapore) were annealed by heating to 95°C for 5 minutes in annealing buffer (500 mM MgCl_2_; 500 mM KCl; 200 mM Tris-HCl, pH 8.0) and left in room temperature to cool down overnight. These were subsequently incubated with the recombinant Zic3 in EMSA buffer (10 mM Tris, pH 8.0; 0.1 mg/ml BSA; 50 µM ZnCl_2_; 100 mM KCL; 0.5 mM MgCl_2_; 10% glycerol, 0.1% SDS; 2 mM β-mercaptoethanol) for 1 hour at 4°C. The reaction was subsequently run on 5% native Tris-Glycine polyacrylamide gel electrophoresis. Gel was scanned in Typhoon Scanner (GE Healthcare, USA). The affinity of protein to DNA was determined by titrating 0–250 nM of protein against 1 nM of annealed probes.

### Morpholino injection and rescue

Zic3 knockdown was performed using a translation-blocking antisense morpholino oligonucleotide (MO) purchased from Gene Tools, LLC (USA). The MO sequence was 5′-AGG TTA GTG GAG TGA ACG GGT ACC G-3′. A standard control antisense MO was also obtained from Gene Tools, LLC with the following sequence 5′-CCT CTT ACC TCA GTT ACA ATT TAT A-3′. For microarray, 1.7 ng Zic3 MO was injected into 1-cell stage embryos. Rescue was performed using 20 pg of *zic3* mRNA without morpholino-binding site. Capped *zic3* mRNA was synthesized using mMessage mMachine Kit (Ambion, USA). Results were obtained from at least three different experiments on embryos from random pairs.

### Microarray hybridization and data analysis

For gene expression profiling, custom made zebrafish oligonucleotide microarray (Agilent Technologies; GIS V2 with some modifications) containing 44,000 oligonucleotide probes (60 mer long; including positive and negative controls designed by Agilent and beta-actin controls) was used. The microarray was performed according to Agilent's One-Color Microarray Based Gene Expression Analysis (Quick Amp Labeling) protocol (Version 5.7, March 2008) and RNA Spike-In-One Color. Arrays were probed using cDNAs reverse transcribed in the presence of Cy3-dUTP using 600 ng of total RNA from either wild-type control or Zic3 knockdown embryos (8 hpf), or from either non zic3-expressing cells or zic3-expressing cells (24 hpf). Labeled cDNA was denatured and hybridized at 42°C for 16 h in a hybridization oven (Agilent Technologies, USA). After hybridization, the slides were washed and scanned for fluorescence detection on Agilent DNA Microarray Scanner. Scanned images were analyzed using Agilent Feature Extraction Software (v10.5.1.1). Feature extracted data were analyzed in Genespring software (Agilent Technologies, USA). Statistically significant gene expression was identified using Significance Analysis of Microarrays (SAM 3.05) for each successive time point [114]. Threshold values were set as follows: q-value<0.8, predicted false discovery rate (FDR)<0.05%. Genes were annotated using the “Unigene & Gene Ontology Annotation Tool” available at GIS site (http://123.136.65.67/). Genes were subjected to pathway assembly using Ingenuity Pathway Analysis (IPA; http://www.ingenuity.com). Selected genes (Fig. 4C; Table S7) were validated using real time RT-PCR (qRT-PCR) by assessing their expression level changes in embryos injected with higher dose of morpholino (3.4 ng) to show similar trend with microarray regulation.

### Enhancer activity assay

Tested genomic regions encompassing the peaks with ∼200 bp flanking sequence at each side were amplified using PCR (primer list in Additional file 5) and cloned into *Sal*I and *Bam*HI sites of the pTol2-GFP reporter vector containing a minimal promoter from the mouse *cFos* gene [115]. Transposase mRNA was synthesized using mMESSAGE mMACHINE T3 Kit (Ambion, USA) and purified using RNeasy Mini Kit (QIAGEN, Germany). A total of 20 pg of the circular reporter plasmid and 50 pg of transposase mRNA were co-injected into 1–2-cell stage embryos. For each construct, two batches of at least 100 embryos were injected and assayed for *egfp* expression at 24 hpf. A consistent *egfp* expression pattern observed in at least 20% of injected embryos was considered as positive. The reporter vector alone showed expression in muscles and blood cells in G_0_ embryos (data not shown). Embryos positive for *egfp* expression were subsequently processed for whole mount immunohistochemistry (IHC) with anti-GFP antibody. qPCR was used to determine *egfp* expression level at 8 hpf since morphological identification of tissue specificity at this stage was difficult.

## Supporting Information

Figure S1 Anti-Zic3 antibody specifically recognizes recombinant and endogenous Zic3 proteins. A, Alignment of C-terminal amino acid sequences of zebrafish Zic proteins. Anti Zic3 antibody was designed to recognize a region (denoted in bold, in boxed area) in the C-terminal of Zic3 protein which is unique, having between 15% to 38% similarity with other Zic family members (highest similarity with Zic2a and Zic4). B, full-length Zic3 recombinant protein containing an MBP tag (Zic3_ORF) was expressed in bacteria and isolated with affinity chromatography. Left panel shows protein SDS-PAGE of uninduced bacterial lysate containing Zic3 expression construct (U), induced bacterial lysate expressing Zic3_ORF (I), eluted lysate of Zic3_ORF (E), and Zic3_ORF treated with TEV protease to remove MBP tag (TEV). Right panel shows Western blot with anti Zic3 primary antibody. Additional lanes show Coomassie blue staining and western blot of a duplicate gel of whole embryonic cell extract (wce), as well as western blot of immunoprecipitated Zic3 protein (ChIP) detected using anti Zic3 primary antibody and a light chain specific anti rabbit IgG secondary antibody. Notice protein bands corresponding to Zic3 with MBP tag (92 KDa, arrow) and without tag (52 KDa, white arrowhead), as well as IgG light chain at 25 KDa. C, mass spectrometry result of the recombinant protein band at 52 kDa (in B, left panel), which matched to zebrafish Zic3 sequence.
(TIF)

Figure S2 Functional categories enriched in different subsets of genes associated with peaks identified at 8 and 24 hpf. List of biological process (A, C, E) and tissue specific expression terms (B, D, F) among genes associated with peaks found at both 8 hpf and 24 hpf (Class I), at 8 hpf only (Class II), and at 24 hpf only (Class III). Light and dark grey bars represent the expected and observed enrichments, respectively, of functional categories indicated along the y-axis. Analysis performed with DAVID algorithm.
(TIF)

Figure S3 EMSA of Zic3 motifs. A, hierarchical clustering of several motif identified from ChIP-seq as well as other motifs known to interact with Zic3. B, EMSA performed using the mouse Zic3 recombinant protein [38] demonstrated cross-species ability of mouse Zic3 protein to recognize the zebrafish motif. C, EMSA with two other motifs identified through ChIP-seq (left panel) and their corresponding mutated versions (right panel).
(TIF)

Figure S4 Defects in cell fate specification and convergent-extension movement in Zic3 knockdown embryos. A, WISH of *sox17a* expression shows the expansion of the mesendoderm territory (marked by dashed line) in Zic3 morphants. B, WISH of *ntla* in the notochord at 8 hpf in control and Zic3 morphants. C–D, WISH of *pax3a* and *sox19a* at 10 hpf to label the neural plate border. Dashed line marks the width of neural plate. E–F, average width of the neural plate was measured at the dashed line in control and Zic3 morphants. Error bars represent standard deviation.
(TIF)

Figure S5 WISH of Zic3 target genes. A, WISH of *rock2b* shows the reduction of expression domain in the dorsal forerunner cells in Zic3 morphant embryos. B, WISH of *dvl2* in control and Zic3 morphant embryos. C, WISH of representative Zic3 target genes at 8 hpf whose expression were downregulated in Zic3 knockdown embryos. Dorsal is to the right.
(TIF)

Figure S6 Effect of Zic3 knockdown in migration of the dorsal forerunner cells. Expression of sox17a (A–B) and foxj1a (C–D) marks the dorsal forerunner cells in controls and Zic3 morphant embryos at 8 hpf.
(TIF)

Figure S7 Enrichment of functional categories of genes associated with proximal and distal Zic3 binding sites. Light and dark grey bars represent the expected and observed enrichments, respectively, of functional categories indicated along the y-axis. A, list of biological processes enriched in genes with peaks beyond −5 kb to +1 kb region. B, a similar enrichment in genes with peaks beyond +/−50 kb from TSS. Analysis performed on DAVID algorithm.
(TIF)

Figure S8 Zic3 is responsible for reporter gene expression in tested enhancers. Enhancer fragments 7-211, 4-16, and 17-24 was either injected alone or co-injected with Zic3 MO into wild-type embryos. GFP expression level in the MO co-injected constructs (MO) was assayed using qRT-PCR and presented as a fraction of that driven by the construct alone (control). A significant decrease in reporter expression when construct was co-injected with MO confirms Zic3-dependent activation of reporter expression through binding to these enhancers.
(TIF)

Table S1 List of validated ChIP-seq peaks of Zic3 binding. Genomic location of some ChIP-seq peaks validated by qPCR including sequences of primer used for qPCR.
(XLS)

Table S2 GO enrichment of ChIP-seq peaks at 8 hpf. Analysis was performed using the DAVID algorithm.
(XLS)

Table S3 List of genes detected by microarray analysis (8 hpf). These genes were differentially regulated according to microarray analysis of transcriptome in Zic3 morphants and control embryos at 8 hpf.
(XLS)

Table S4 GO enrichment of microarray genes (8 hpf).
(XLS)

Table S5 Genes with ChIP-seq peak at 8 hpf. List of genes expressed differentially at 8 hpf with at least one ChIP-seq peak within 100 kb of their TSS.
(XLS)

Table S6 GO enrichment of microarray genes with ChIP-seq peak at 8 hpf. List of enriched GO terms among genes differentially regulated in microarray and associated with at least one peak in ChIP-seq (Zic3 target genes) at 8 hpf.
(XLS)

Table S7 qRT-PCR of selected target genes. qRT-PCR measurement of gene expression changes as a result of Zic3 knockdown. Expression was assayed in zebrafish embryos injected with either 1.7 ng and 3.4 ng or 3.4 ng alone of Zic3 MO.
(XLSX)

Table S8 List of genes detected by microarray analysis (24 hpf). List of genes which are enriched or de-enriched in *zic3*- expressing cells of the neural tube obtained by FACS at 24 hpf. Fold change was calculated by comparing expression levels in *zic3*-expressing cells to that in cells negative for *zic3* expression.
(XLS)

Table S9 GO enrichment of microarray genes (24 hpf). List of GO terms enriched among genes co-expressed with *zic3* at 24 hpf.
(XLS)

Table S10 List of microarray genes with peak (24 hpf). List of differentially expressed genes containing at least one ChIP-seq peak within 100 kb of their TSS at 24 hpf.
(XLS)

Table S11 GO enrichment of microarray genes with ChIP-seq peaks (24 hpf). GO terms enriched among genes co-expressed with *zic3* and associated with at least one ChIP-seq peak at 24 hpf.
(XLS)

Table S12 List of tested enhancers. List of selected enhancers tested using *in vivo* enhancer assay with their genomic locations, primer sequences, and flanking genes.
(XLS)

Table S13 List of ChIP-seq peaks (8 hpf). List of genome-wide Zic3 binding sites identified by ChIP-seq at 8 hpf.
(XLS)

Table S14 List of ChIP-seq peaks (24 hpf). List of genome-wide Zic3 binding sites identified by ChIP-seq at 24 hpf.
(XLS)

Text S1 Supplementary experimental methods. Description of additional methods used in the study.
(DOC)
