# Supplementary figures and images for: Genome Wide Analysis Reveals Zic3 Interaction with Distal Regulatory Elements of Stage Specific Developmental Genes in Zebrafish
Source: PLoS Genet. 2013 Oct 31;9(10):e1003852. doi: 10.1371/journal.pgen.1003852 (PMC3814314; doi:10.1371/journal.pgen.1003852)

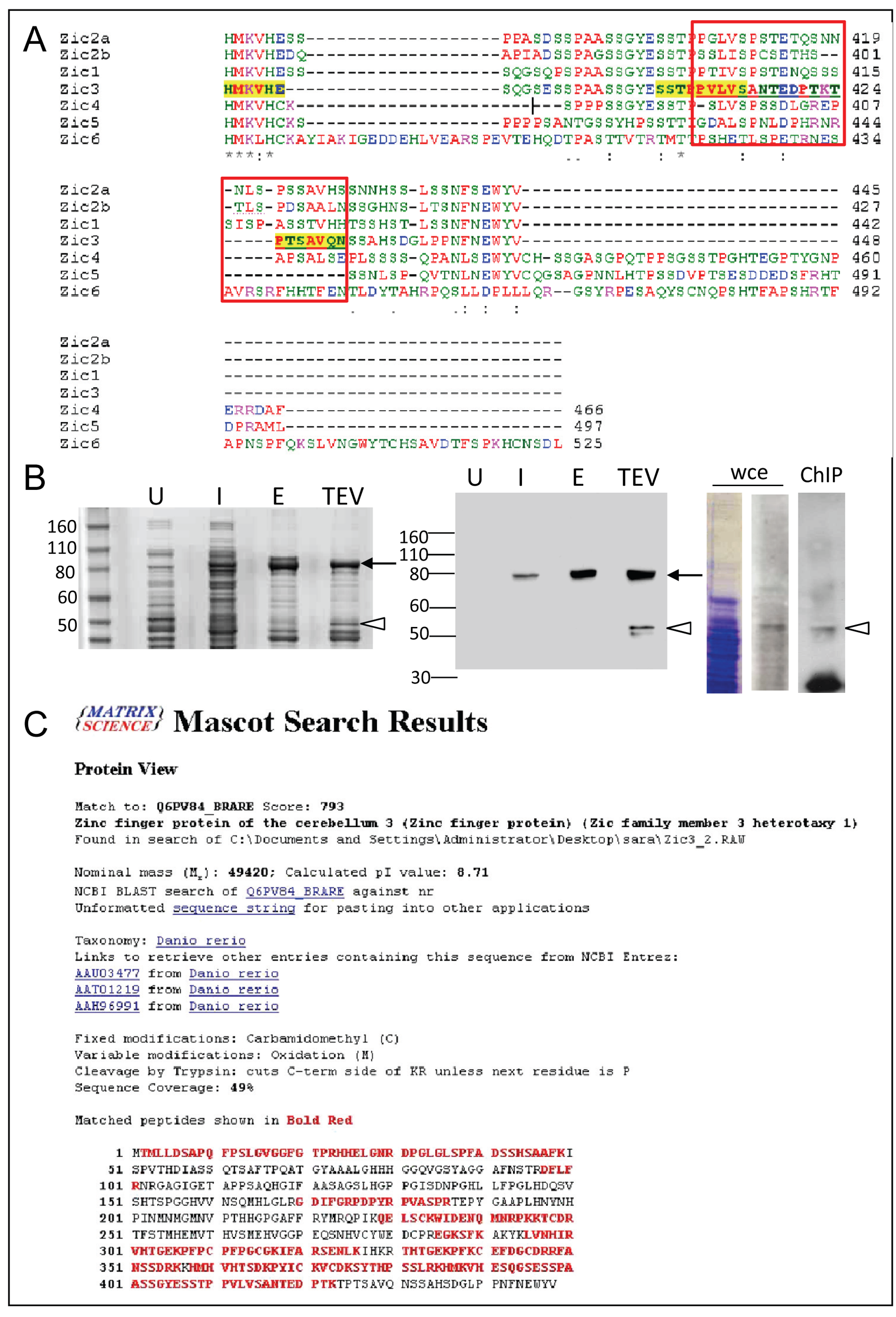

Supplement: Figure S1 — Anti-Zic3 antibody specifically recognizes recombinant and endogenous Zic3 proteins. A, Alignment of C-terminal amino acid sequences of zebrafish Zic proteins. Anti Zic3 antibody was designed to recognize a region (denoted in bold, in boxed area) in the C-terminal of Zic3 protein which is unique, having between 15% to 38% similarity with other Zic family members (highest similarity with Zic2a and Zic4). B, full-length Zic3 recombinant protein containing an MBP tag (Zic3_ORF) was expressed in bacteria and isolated with affinity chromatography. Left panel shows protein SDS-PAGE of uninduced bacterial lysate containing Zic3 expression construct (U), induced bacterial lysate expressing Zic3_ORF (I), eluted lysate of Zic3_ORF (E), and Zic3_ORF treated with TEV protease to remove MBP tag (TEV). Right panel shows Western blot with anti Zic3 primary antibody. Additional lanes show Coomassie blue staining and western blot of a duplicate gel of whole embryonic cell extract (wce), as well as western blot of immunoprecipitated Zic3 protein (ChIP) detected using anti Zic3 primary antibody and a light chain specific anti rabbit IgG secondary antibody. Notice protein bands corresponding to Zic3 with MBP tag (92 KDa, arrow) and without tag (52 KDa, white arrowhead), as well as IgG light chain at 25 KDa. C, mass spectrometry result of the recombinant protein band at 52 kDa (in B, left panel), which matched to zebrafish Zic3 sequence. (TIF) [file pgen.1003852.s001.tif]

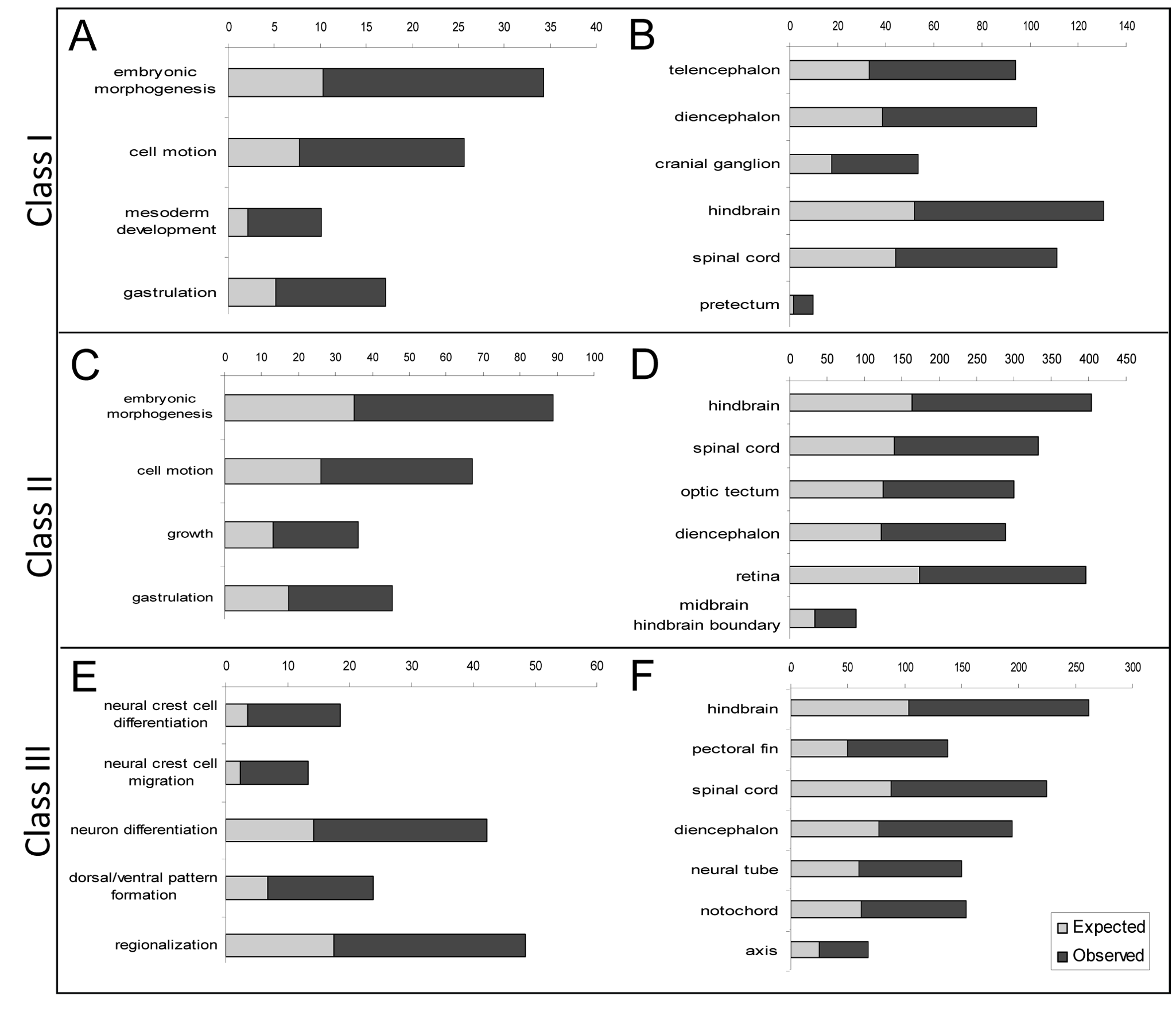

Supplement: Figure S2 — Functional categories enriched in different subsets of genes associated with peaks identified at 8 and 24 hpf. List of biological process (A, C, E) and tissue specific expression terms (B, D, F) among genes associated with peaks found at both 8 hpf and 24 hpf (Class I), at 8 hpf only (Class II), and at 24 hpf only (Class III). Light and dark grey bars represent the expected and observed enrichments, respectively, of functional categories indicated along the y-axis. Analysis performed with DAVID algorithm. (TIF) [file pgen.1003852.s002.tif]

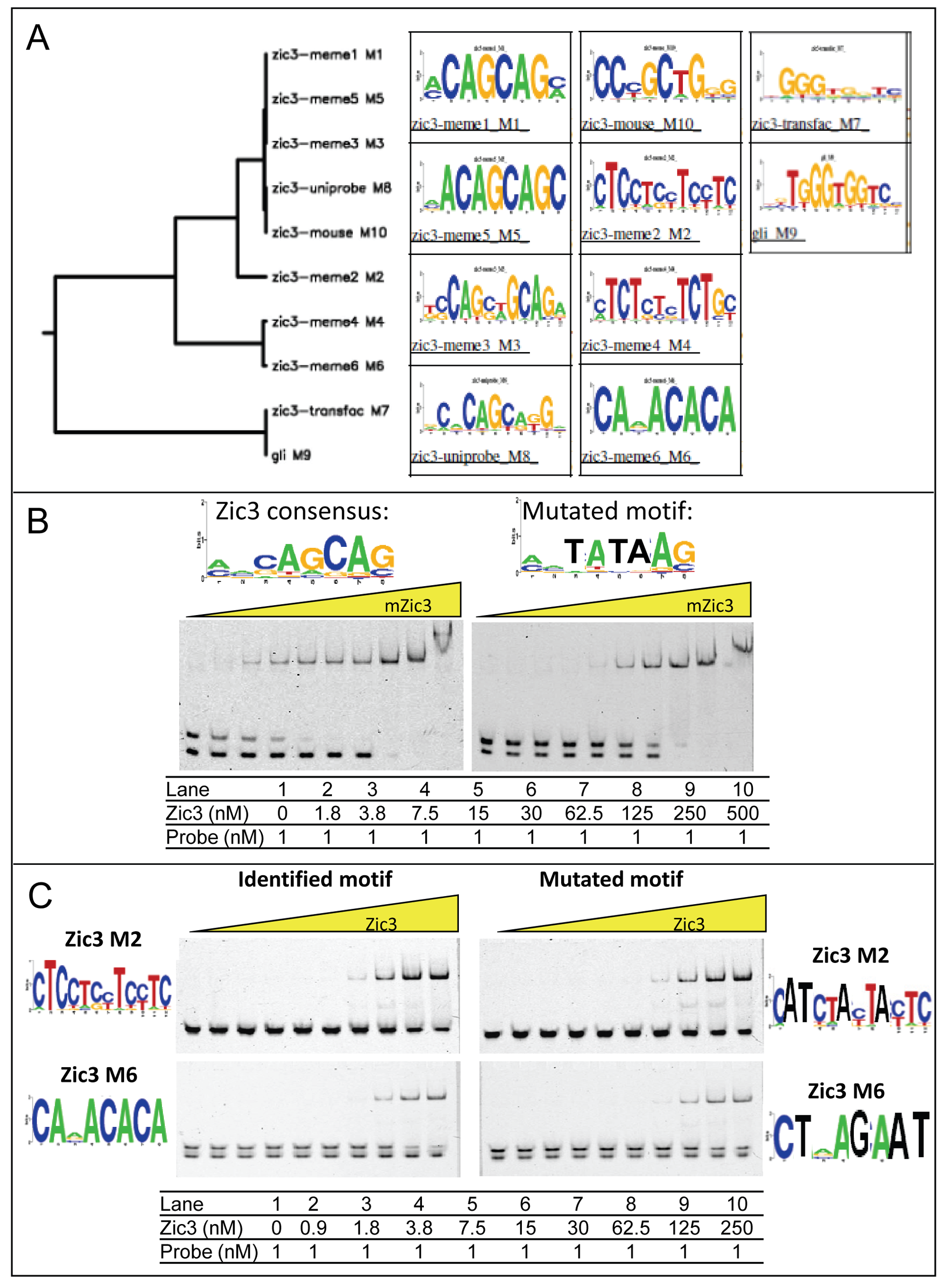

Supplement: Figure S3 — EMSA of Zic3 motifs. A, hierarchical clustering of several motif identified from ChIP-seq as well as other motifs known to interact with Zic3. B, EMSA performed using the mouse Zic3 recombinant protein [38] demonstrated cross-species ability of mouse Zic3 protein to recognize the zebrafish motif. C, EMSA with two other motifs identified through ChIP-seq (left panel) and their corresponding mutated versions (right panel). (TIF) [file pgen.1003852.s003.tif]

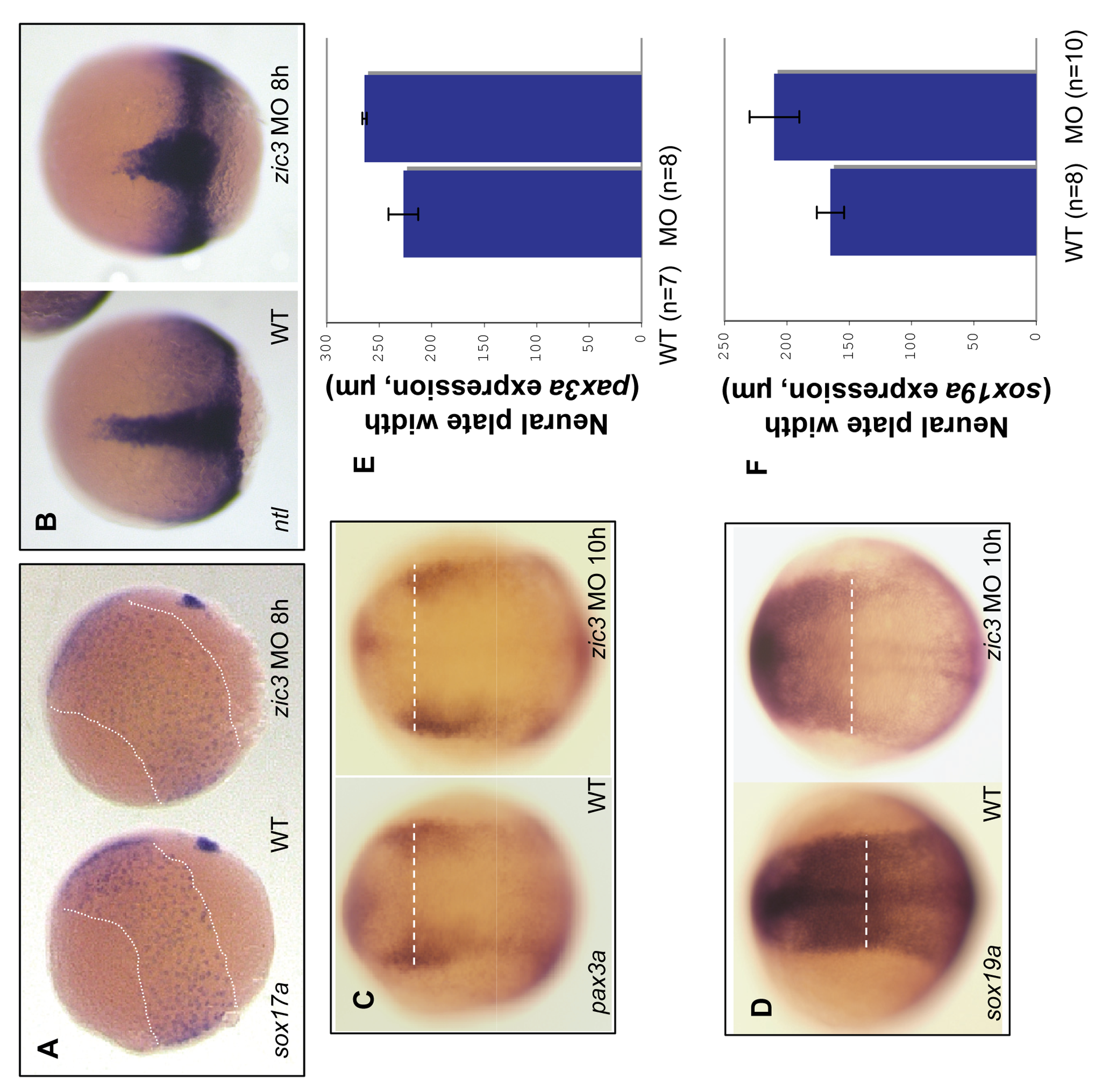

Supplement: Figure S4 — Defects in cell fate specification and convergent-extension movement in Zic3 knockdown embryos. A, WISH of sox17a expression shows the expansion of the mesendoderm territory (marked by dashed line) in Zic3 morphants. B, WISH of ntla in the notochord at 8 hpf in control and Zic3 morphants. C–D, WISH of pax3a and sox19a at 10 hpf to label the neural plate border. Dashed line marks the width of neural plate. E–F, average width of the neural plate was measured at the dashed line in control and Zic3 morphants. Error bars represent standard deviation. (TIF) [file pgen.1003852.s004.tif]

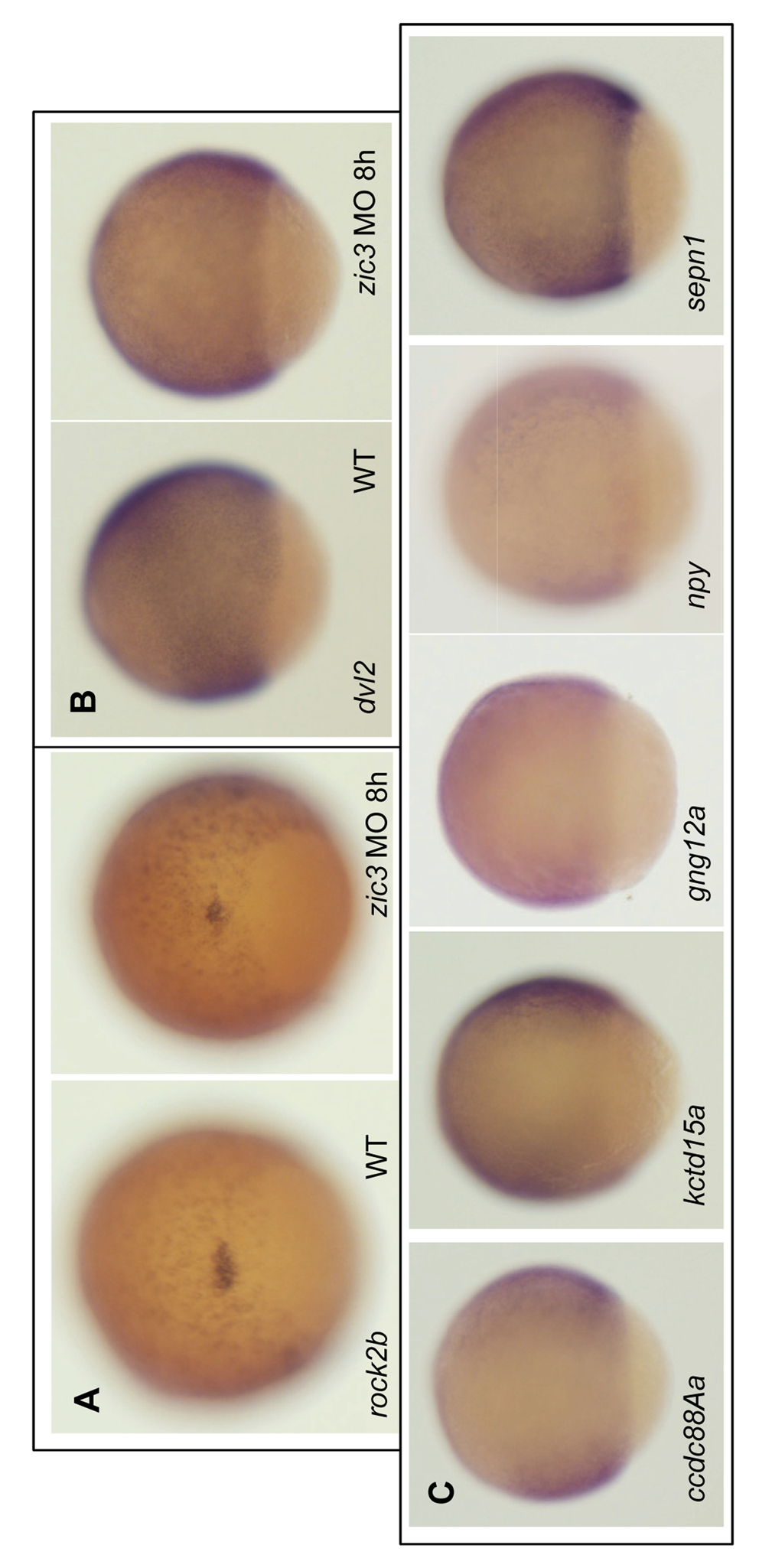

Supplement: Figure S5 — WISH of Zic3 target genes. A, WISH of rock2b shows the reduction of expression domain in the dorsal forerunner cells in Zic3 morphant embryos. B, WISH of dvl2 in control and Zic3 morphant embryos. C, WISH of representative Zic3 target genes at 8 hpf whose expression were downregulated in Zic3 knockdown embryos. Dorsal is to the right. (TIF) [file pgen.1003852.s005.tif]

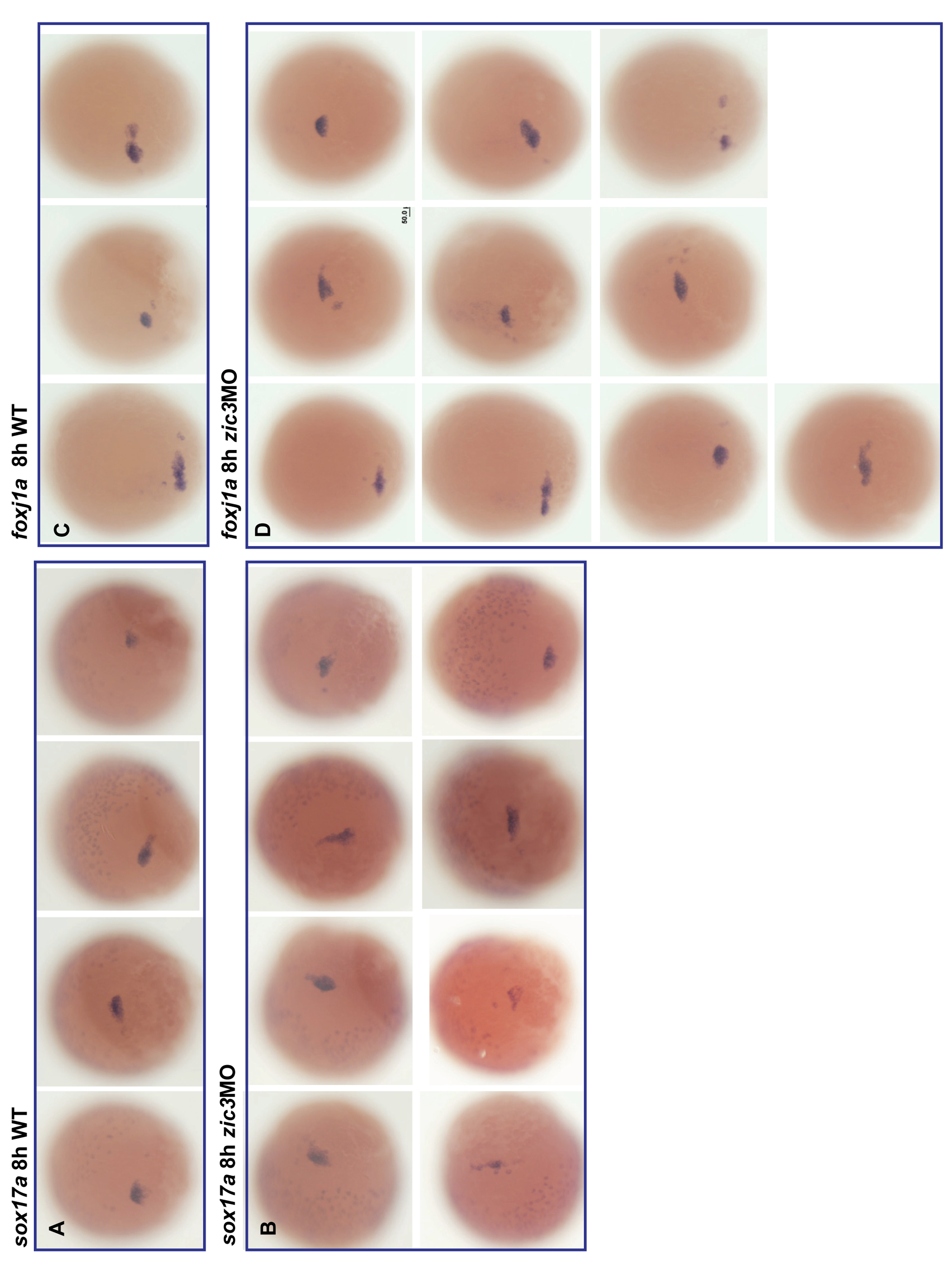

Supplement: Figure S6 — Effect of Zic3 knockdown in migration of the dorsal forerunner cells. Expression of sox17a (A–B) and foxj1a (C–D) marks the dorsal forerunner cells in controls and Zic3 morphant embryos at 8 hpf. (TIF) [file pgen.1003852.s006.tif]

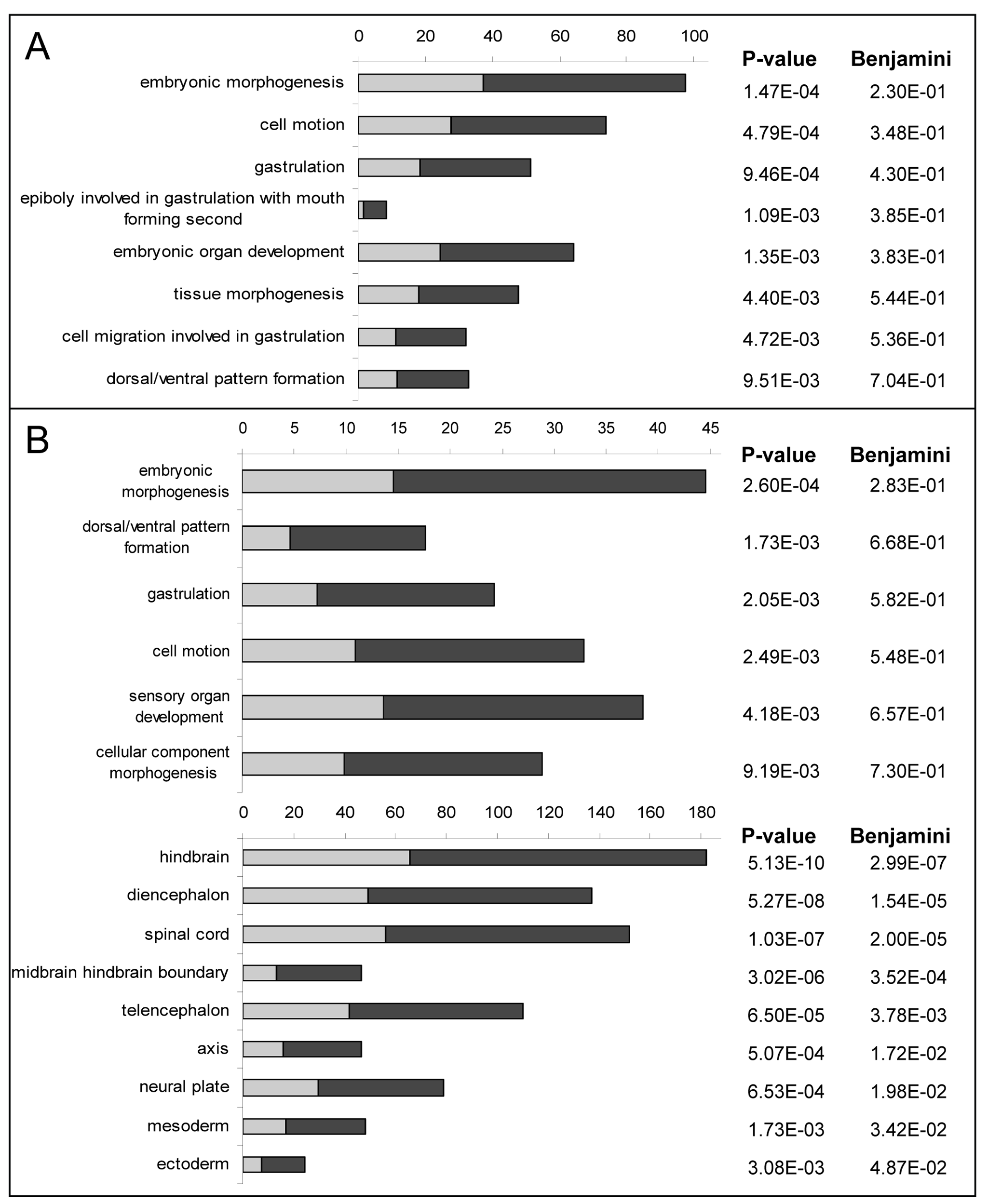

Supplement: Figure S7 — Enrichment of functional categories of genes associated with proximal and distal Zic3 binding sites. Light and dark grey bars represent the expected and observed enrichments, respectively, of functional categories indicated along the y-axis. A, list of biological processes enriched in genes with peaks beyond −5 kb to +1 kb region. B, a similar enrichment in genes with peaks beyond +/−50 kb from TSS. Analysis performed on DAVID algorithm. (TIF) [file pgen.1003852.s007.tif]

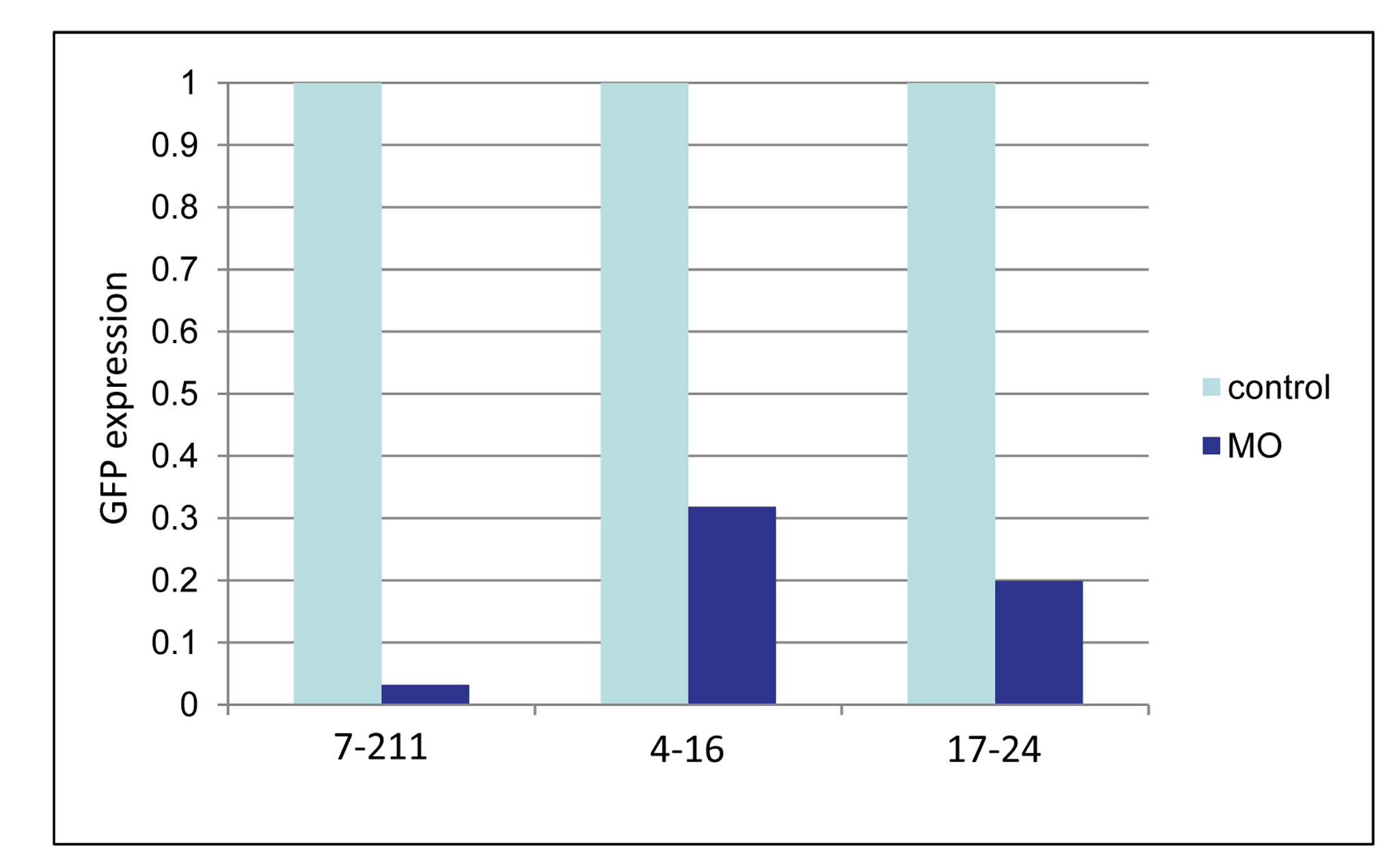

Supplement: Figure S8 — Zic3 is responsible for reporter gene expression in tested enhancers. Enhancer fragments 7-211, 4-16, and 17-24 was either injected alone or co-injected with Zic3 MO into wild-type embryos. GFP expression level in the MO co-injected constructs (MO) was assayed using qRT-PCR and presented as a fraction of that driven by the construct alone (control). A significant decrease in reporter expression when construct was co-injected with MO confirms Zic3-dependent activation of reporter expression through binding to these enhancers. (TIF) [file pgen.1003852.s008.tif]
