# Supplementary material for: Genome Wide Analysis Reveals Zic3 Interaction with Distal Regulatory Elements of Stage Specific Developmental Genes in Zebrafish
Source: PLoS Genet. 2013 Oct 31;9(10):e1003852. doi: 10.1371/journal.pgen.1003852 (PMC3814314; doi:10.1371/journal.pgen.1003852)
Supplement: Text S1 — Supplementary experimental methods. Description of additional methods used in the study. (DOC) [file pgen.1003852.s023.doc]

**Supplementary Methods**

**Chromatin Immunoprecipitation**

Antibodies against zebrafish Zic3 protein (Mimotopes, Australia) was raised against a highly antigenic region at the C-terminal region (amino acids 411-431) which is non-conserved among members of the Zic protein family (Fig. S1A). Specificity of the antibody was confirmed by its ability to recognize full-length Zic3 recombinant protein (Zic3_ORF, see below), whose identity was further confirmed by mass spectrometry (Fig. S1B, C). The ability of the antibody to recognize native Zic3 protein was demonstrated through western blot of whole cell extract from zebrafish embryos (Fig. S1B), and the ability of the antibody to pull down Zic3 protein was demonstrated by western blot using the same Zic3 primary antibody and a light chain specific anti rabbit IgG (Jackson Immunoresearch, USA) as secondary antibody. Chromatin Immunoprecipitation (ChIP) was performed according to a published protocol (Wardle et al., 2006) with an addition of a deyolking step according to Link and colleagues (2006), with modifications. Briefly, embryos were transferred to cold deyolking buffer (55 mM NaCl, 1.8 mM KCl, 1.25 mM NaHCO_3_) using 200 µl pipette tips, shaken by inverting tube several times, and centrifuged at 300 × g for 1 minute. The pellet was washed once with PBS and subsequently fixed in 1.1% formaldehyde containing 5 mM Hepes-KOH pH 7.5, 10 mM NaCl, 0.1 mM EDTA, and 0.05 mM EGTA before lysis and sonication. Fixed FACS-sorted cell were directly lysed and sonicated. Pull down was performed by incubating sonicated chromatin for 5 hours at 4°C with Dynal Protein G magnetic beads (Invitrogen, USA) coated with 10 µg of antibody, after which beads were washed 7 times with RIPA buffer (500 mM LiCl, 50 mM Hepes-KOH pH 7.6, 1 mM EDTA, 0.7% Na-Deoxycholate, 1% Igepal-CA-630) and de-crosslinked overnight in elution buffer (50 mM Tris-HCl pH 8.0, 10 mM EDTA, 1% SDS) at 65°C before DNA isolation using phenol-chloroform-isoamyl alcohol (Ambion, USA). ChIP DNA was sequenced on the Illumina Genome Analyzer (Illumina, USA) with single end, 35bp reads. Library preparation was performed using the Illumina ChIP-seq sample preparation kit (Cat. No. IP-102-1001, Illumina, USA) according to the manufacturer’s protocol.

**Whole mount *in situ* hybridization (WISH)**

WISH was performed as previously described with minor modifications of buffer composition. Briefly, hybridization buffer contained 5% of dextran sulfate and 0.5x detection buffer contained 2% of polyvinyl alcohol (Aldrich, USA). Full-length cDNA of *zic3* (GenBank: AY576007) was amplified using the following primers: 5'-AGC TTA CGT GAA ATT GCG CTC-3' and 5’-CTC CAC CTG AAA ACG GAC TTG-3'. Resulting PCR product was cloned into pGEM-TEasy vector (Promega). Digoxigenin (DIG)-labeled RNA probes antisense to *zic3* and *egfp* were synthesized using a DIG labeling mix (Roche) and MEGAscript SP6 or T7 kit (Ambion). Images were captured using Leica M205FA stereomicroscope (Leica, Germany).

**Real time quantitative PCR (qPCR)**

qPCR was performed using Maxima® SYBR Green qPCR Master Mix (Fermentas, Lithuania) on the ABI 7500 Real-time PCR system according to manufacturer’s instructions. Using input DNA as a reference, fold change was calculated by normalizing Ct values in ChIP and input samples against a negative control region using the 2^-∆∆Ct^ method .

**Extraction of total RNA**

Total RNA was extracted from whole embryos using Trizol reagent (Invitrogen) according to the manufacturer's instructions. Control RNA samples were extracted from wild type zebrafish embryos and used as reference for labeling and probing microarrays. The integrity of RNA samples was determined using Agilent RNA 6000 Nano chip and size separated using Agilent 2100 Bioanalyzer. The RNA concentrations were determined using Nanodrop (Thermo Scientific, USA).

**Whole mount immunohistochemistry**

For whole mount IHC embryos were dechorionated and fixed overnight in Histochoice (Amresco, USA) at 4°C. Briefly, the fixed embryos were treated 10 min with 3% H_2_O_2_ in PBS and permeabilized 10 min with 0.1% Triton X-100 in PBS containing 0.1% sodium citrate. Embryos were incubated overnight with primary antibody at 4^0^C and then within 4 hrs with HRP-conjugated secondary antibody (1:500, Molecular Probes) at room temperature. The following primary antibodies were used: polyclonal anti-GFP antibody (1:250, Living Colors, Clontech, USA) and monoclonal anti-myosin (sarcomere) antibody (1:100, MF20, Developmental Studies Hybridoma Bank). Labeling was detected with diaminobenzidine as a substrate. Embryos were washed and mounted in 50% glycerol in PBS for imaging.

**Supplemental References**

1. Lim LS, Hong FH, Kunarso G, Stanton LW (2010) The pluripotency regulator Zic3 is a direct activator of the Nanog promoter in ESCs. Stem Cells 28: 1961-1969.

2. Korzh V, Sleptsova I, Liao J, He J, Gong Z (1998) Expression of zebrafish bHLH genes ngn1 and nrd defines distinct stages of neural differentiation. Dev Dyn 213: 92-104.

3. Livak KJ, Schmittgen TD (2001) Analysis of relative gene expression data using real-time quantitative PCR and the 2(-Delta Delta C(T)) Method. Methods 25: 402-408.

4. Cast AE, Gao C, Amack JD, Ware SM (2012) An essential and highly conserved role for Zic3 in left-right patterning, gastrulation and convergent extension morphogenesis. Dev Biol 364: 22-31.
